# Amygdala-hippocampal dynamics during salient information processing

**DOI:** 10.1038/ncomms14413

**Published:** 2017-02-08

**Authors:** Jie Zheng, Kristopher L. Anderson, Stephanie L. Leal, Avgusta Shestyuk, Gultekin Gulsen, Lilit Mnatsakanyan, Sumeet Vadera, Frank P. K. Hsu, Michael A. Yassa, Robert T. Knight, Jack J. Lin

**Affiliations:** 1 Department of Biomedical Engineering, University of California, Irvine, California 92697, USA; 2 Helen Wills Neuroscience Institute, University of California, Berkeley, California 94720, USA; 3 Department of Psychology, University of California, Berkeley, California 94720, USA; 4 Department of Neurobiology and Behavior, University of California, Irvine, California 92697, USA; 5 Comprehensive Epilepsy Program, Department of Neurology, University of California, Irvine, California 92868, USA; 6 Department of Neurological Surgery, University of California, Irvine, California 92868, USA

## Abstract

Recognizing motivationally salient information is critical to guiding behaviour. The amygdala and hippocampus are thought to support this operation, but the circuit-level mechanism of this interaction is unclear. We used direct recordings in the amygdala and hippocampus from human epilepsy patients to examine oscillatory activity during processing of fearful faces compared with neutral landscapes. We report high gamma (70–180 Hz) activation for fearful faces with earlier stimulus evoked onset in the amygdala compared with the hippocampus. Attending to fearful faces compared with neutral landscape stimuli enhances low-frequency coupling between the amygdala and the hippocampus. The interaction between the amygdala and hippocampus is largely unidirectional, with theta/alpha oscillations in the amygdala modulating hippocampal gamma activity. Granger prediction, phase slope index and phase lag analysis corroborate this directional coupling. These results demonstrate that processing emotionally salient events in humans engages an amygdala-hippocampal network, with the amygdala influencing hippocampal dynamics during fear processing.

## Abstract (web summary)

Amygdala and hippocampus are involved in processing motivationally salient stimuli but the precise circuit dynamics of the interaction is not understood. Here the authors show that in response to fearful faces in humans, theta/alpha oscillations in the amygdala modulate hippocampal activity dynamics.

Swift detection of social, emotional or threatening stimuli is critical for adaptive fitness in humans. When we interact with each other, emotionally salient stimuli, such as fearful facial expressions, provide ecologically relevant signals that focus our attention towards perceptually relevant information. Thus, recognizing motivationally salient information constitutes an important social and biologically meaningful incentive and plays a key role in guiding our interpersonal behaviour^1^.

Successful detection of and response to motivationally important stimuli have been shown to rely on activity within two brain structures—the amygdala and hippocampus. In particular, the amygdala is critical for prioritizing salient information such as emotion^2^, valence^3^ and motivation^4^. The hippocampus is thought to be important for contextual modulation of fear^5^, emotion judgment^6^ and emotional memory^7^—all operations that are critical for remembering motivationally salient stimuli.

It is commonly assumed that the amygdala exerts directional influence onto the hippocampus during processing of salient information^8^. This network model of salience processing is primarily based on rodent data. For example, the amygdala receives direct subcortical inputs thought to facilitate rapid detection of salient information^9^, consistent with a proposed role of the amygdala in early cognitive engagement that may influence subsequent hippocampal mnemonic processing. Several studies also indicate that manipulating amygdala function alters hippocampal processing of salient information^10^,^11^,^12^. Evidence for this directional influence in humans has only been indirectly inferred from behaviour^13^ and neuroimaging^14^ studies showing that memory enhancement for emotionally arousing stimuli is positively associated with markers of endogenous norepinephrine release from the basolateral amygdala (BLA)^15^. However, there is no direct electrophysiological evidence for amygdala-hippocampal connectivity in humans and thus their directional relationship is unknown.

We addressed this question by presenting salient (dynamic fearful faces) and neutral (landscapes) stimuli to patients with medication resistant epilepsy in whom stereotactic electrodes had been implanted in the amygdala and hippocampus for pre-surgical evaluation. First, we hypothesized that high gamma (HG; 70–180 Hz) band activity (a spatially precise measure of neuronal spiking^16^) will occur earlier in the amygdala than in the hippocampus, consistent with a directional relationship. We next examined the electrophysiological evidence for connectivity between the amygdala and hippocampus. Low-frequency oscillations (theta=4–7 Hz and alpha=8–12 Hz) are ubiquitous in the human hippocampus^17^ and amygdala^18^; fear conditioning studies in rodents suggest that they provide a temporal window for inter-regional communication^19^. Therefore, we hypothesized that low-frequency oscillations mediate functional connectivity between the amygdala and hippocampus by coupling spiking activity in the hippocampus (as indexed by the HG signal) to low-frequency oscillations in the amygdala. Finally, consistent with the model of detection/prioritization by the amygdala and post-detection processing by the hippocampus^10^,^11^,^12^, we hypothesized that the synchronous activity in these two regions would be biased such that it is more likely that the amygdala exerts directional influence on the hippocampus rather than the reverse.

In this study, we show that the amygdala and hippocampus are both engaged in the early stages of salience processing with increased intraregional HG activity and enhanced inter-regional low-frequency synchrony when attending to aversive compared with neutral stimuli. The coupling between these two regions is predominantly unidirectional, with low-frequency oscillations in the amygdala entraining hippocampal HG activity. Overall, these results provide evidence for a directional influence from the amygdala to the hippocampus during processing of motivationally salient stimuli.

## Results

### Experiment design and electrode localization

**Figure 1:**
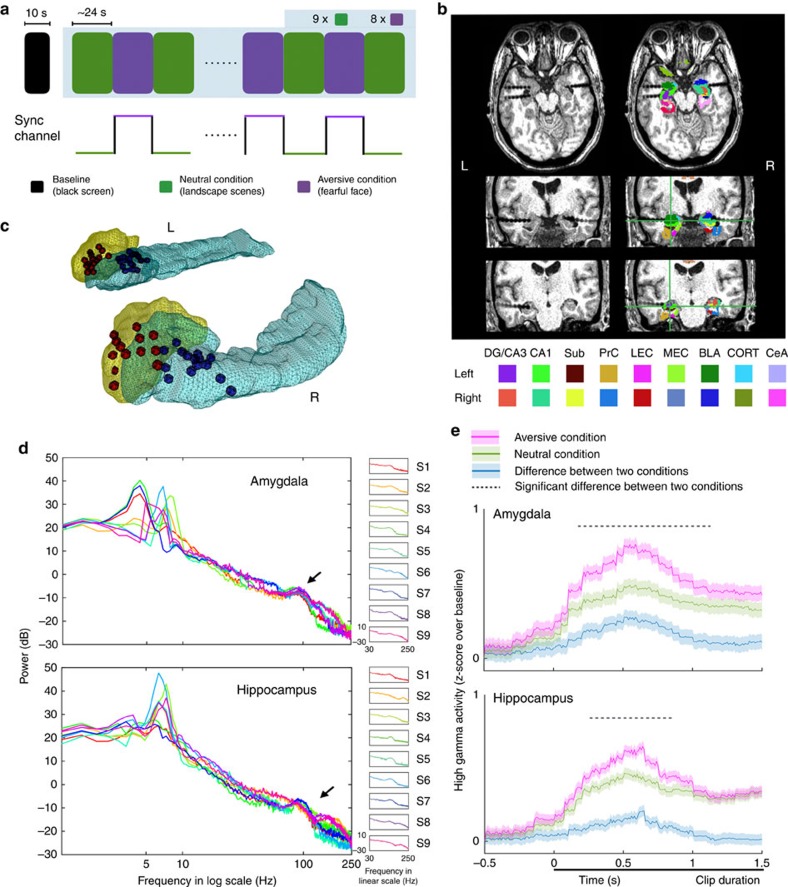
Task, electrode locations, power spectral density and high gamma activity. (**a**) Participants watched silent movie clips consisting of alternating blocks of neutral (landscapes) and aversive stimuli (fearful faces). (**b**) Example MRI and template for a single subject. Electrodes were localized in each participant using co-registered pre-implantation and post-implantation structural T1-weighted MRI scans. A high-resolution template of the hippocampal subfields and amygdala nuclei was aligned to each participant's pre-implantation scan to visualize electrode locations in subject-specific anatomical space. Regions of interest (ROIs) in the medial temporal lobe included the DG/CA3, CA1, subiculum (Sub), perirhinal cortex (PrC), lateral and medial entorhinal cortex (LEC, MEC), parahippocampal cortex (PhC), BLA, CeA and the CORT. Each electrode location was determined by selecting the centre of the electrode (indicated by cross-hairs) and determining which ROI best encompassed the centre of the electrode. (**c**) Electrode localization of all subjects, rendered onto a three-dimensional amygdala and hippocampus model based on the high-resolution template. Red dots indicate electrodes in the amygdala; blue dots denote electrodes in the hippocampus. (**d**) Power spectral density (PSD) in log scale for the amygdala (upper panel) and hippocampus (lower panel). Peaks within theta (4–7 Hz)/alpha (8–12 Hz) and high gamma range (70–180 Hz) were consistently observed in all subjects. The black arrow denotes the power peak in the high gamma range, which is also shown for each individual subject on a linear scale (30–250 Hz) in the small plots. (**e**) High gamma amplitude (70–180 Hz), averaged across participants (± s.e.m. shown as shading around the mean trace) and locked to stimulus onset, is shown for electrodes located in the amygdala and hippocampus (DG/CA3+CA1). Dotted lines represent significant differences between the aversive and neutral condition. (permutation test, see methods). L, left; R, right.

We recorded oscillatory activity in local field potentials (LFPs) from nine human participants with intracranial depth electrodes implanted into the amygdala and the hippocampus. Electro-oculogram (EOG) electrodes and an eye tracker were used for one subject to evaluate the potential influence of saccadic muscle movements on neural signals. We examined neuronal responses while individuals watched aversive movie clips containing blocks of dynamic fearful faces and neutral movie clips of landscapes (Fig. 1a). We employed dynamic fearful faces as a form of aversive stimuli, rather than static facial expressions, to provide participants with temporal cues that mimic real-life social exchanges^20^. The localization of depth electrodes was determined based on co-registered pre- and post-implantation magnetic resonance imaging (MRI), as well as registration to a high-resolution anatomical atlas, labelled with medial temporal lobe regions of interest. Localization of each electrode was performed in a semi-automated manner, guided by the anatomical atlas and visually checked by an experienced rater (S.L.L.). In all subjects, there were two to three depth electrodes located in the BLA and one to three electrodes located in the hippocampus (dentate gyrus (DG)/CA3 or CA1, Fig. 1b and Supplementary Fig. 1). A three-dimensional rendering of the amygdala and hippocampus showed that for all subjects, the electrodes were located in the basal aspects of the amygdala and the anterior hippocampus (Fig. 1c).

### Local power and event-related potentials

Neuronal networks typically demonstrate activity in several oscillatory bands that cover both low- and high-frequency spectra with distinct roles in neuronal communication^21^. Whereas high gamma band activity is a spatially precise measure of local neuronal population spiking^16^, temporal synchronization of low-frequency phase is thought to mediate inter-regional communication^22^. Therefore, we first determined the spectral specificity of low- and high-frequency oscillations in LFP. The power spectral density (PSD) plots revealed that each subject had a specific frequency peak in the theta/alpha and high gamma frequency ranges (Fig. 1d). These peaks are thought to reflect coherent oscillatory processes^23^. We then band passed the raw LFP signal to extract separate frequency components. These analyses showed that the low frequency of the amygdala and high gamma band power envelope of the hippocampus tended to co-occur in time during the aversive condition (Supplementary Fig. 2). Additional analyses demonstrated that event-related potentials (ERP; Supplementary Fig. 3) and ocular muscle activity (Supplementary Figs 4 and 5a,b) did not contaminate the neural signals used in subsequent analyses.

### Amygdala and hippocampus high gamma tracks salient stimuli

We explored the temporal profile of the oscillatory response to fearful faces versus landscapes. Electrodes localized in the amygdala (BLA) and hippocampus (DG/CA3+CA1) with high-resolution MRIs as shown in Fig. 1c were included in the analysis. We then focused on the temporally resolved changes in HG amplitude and examined the coordinated timing of amygdala and hippocampus neuronal responses during the processing of aversive compared with neutral stimuli (Fig. 1e). The onset time was defined as the earliest time point at which two conditions showed a significant difference in HG amplitudes; the peak time was defined as the latency of the maximum magnitude of differences in HG amplitudes between the conditions. The average HG amplitude across trials was higher for the aversive relative to the neutral condition after 123±18 ms (mean±s.e.m.) in the amygdala and after 241±22 ms in the hippocampus post-stimulus onset (onset time, *t*-test, *P*<0.05). Similarly, HG activity peaked earlier in the amygdala compared with the hippocampus (amygdala=493±31 ms versus hippocampus=641±42 ms; peak time, *t*-test, *P*<0.05). These findings indicate that the amygdala and hippocampus are both engaged in the early stages of salience processing, with amygdala activation preceding hippocampal activation.

### Amygdala-hippocampal low-frequency coupling

**Figure 2:**
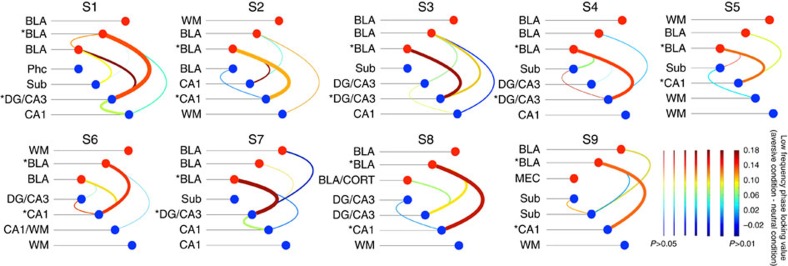
Differences in amygdala-hippocampal low-frequency phase coupling. Low-frequency phase coupling differences (aversive—landscapes) for pairs of electrodes targeting the amygdala (red dots) and hippocampal subfields (blue dots), depicted with hive plots. Differences in the PLV between aversive and neutral conditions are presented in colour, with warmer colours indicating a greater magnitude of the contrast. Significance levels derived from permutation testing are indicated by the thickness of lines connecting each electrode pair. Asterisks represent electrode pairs with the most significant PLV that were used for directional coupling analyses in Figs 3 and 4.

Given the distinct HG temporal profiles of the amygdala and hippocampus, we then investigated whether these two regions interact through low-frequency phase coupling. The inter-regional coordination was examined with phase locking values (PLV) as a metric of effective connectivity between electrodes targeting the amygdala (red) and hippocampus (blue, Fig. 2). The three most medial electrodes targeting the amygdala and four most medial electrodes targeting the hippocampus were included in the phase coupling analysis. Note that this selection criterion was agnostic with regard to the experimental conditions (that is, aversive versus neutral) and the magnitude of phase coupling. Because of individual differences in the frequency of the event-related low-frequency band, we first selected a specific band for each subject centred on the low-frequency peak in the PSD (amygdala=6.02±0.49 Hz; hippocampus=6.51±0.16 Hz, mean±s.e.m., Fig. 1d) with a bandwidth of 4 Hz (ref. 24). We characterized the timing relationships of the low-frequency phase among all electrode pairs for each subject and calculated the PLV within and between the aversive and neutral conditions. Within each condition, amygdala-hippocampal PLVs were significantly increased compared with the null distribution (all *P*<0.05, permutation test, Supplementary Fig. 6). We next tested PLV differences across conditions and found enhanced PLV between the low-frequency phases of BLA and the low-frequency phases of DG/CA3 as well as CA1 hippocampal subfields while viewing aversive compared with neutral movie clips (all *P*<0.05, permutation test, Fig. 2).

To validate the role of low-frequency phase coupling in coordinating amygdala-hippocampal network communication during processing of motivationally salient information, we examined the PLV spectra between the most significant phase coupling electrode pairs (denoted by asterisks in Fig. 2). Across all subjects, the PLV increased when viewing aversive fearful faces compared with neutral stimuli (main effect: F (1,67)=8.88, *P*=0.004) and peaked in the low-frequency band for BLA-CA1 and BLA-CA3 compared with BLA- parahippocampal and BLA- subiculum electrode pairs (Supplementary Fig. 7a). Further, the magnitude of low-frequency PLV varied among hippocampal sub-regions (F (3,67)=2.88, *P*=0.042) with the highest PLV observed for the BLA-CA1 electrode pairs. The phase coupling between BLA and CA1/CA3 were significantly greater than the other hippocampal sub-regions (*t*-test, *P*<0.05; Supplementary Fig. 7b). These findings indicate that the amygdala and CA1/CA3 regions of the hippocampus exhibit strong low-frequency synchrony during processing of aversive stimuli.

### Directional amygdala-hippocampus phase-amplitude coupling

**Figure 3:**
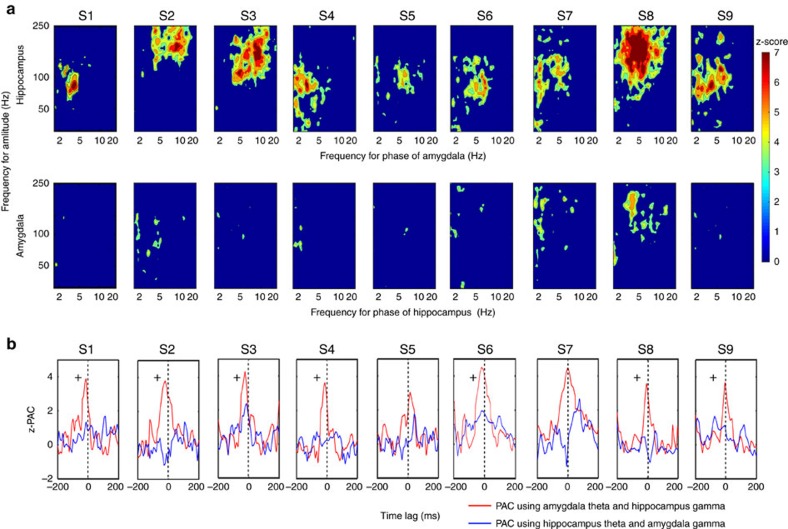
Amygdala-hippocampus directional PAC and phase lag analysis. (**a**) PAC comodulogram for differences between the aversive and the neutral conditions is shown, with warmer colours denoting higher *z*-scores. The high gamma amplitude in the hippocampus was phase-locked to the phase of amygdala low-frequency (theta and alpha) rhythms (all *P*<0.01, permutation test). In contrast, the reciprocal directional PAC modulation (for example, high gamma amplitude in the amygdala phase-locked to phase of hippocampal low-frequency activity) was nearly absent. (**b**) Phase lag analysis. The PAC modulation index between low-frequency phase and high gamma amplitude was estimated across time lags for the aversive condition. The red line represents the *z*-PAC between subject-specific amygdala low-frequency phase and hippocampal high gamma amplitude (70–180 Hz). The blue line denotes the *z*-PAC between hippocampus low-frequency phase and amygdala high gamma amplitude. Cross (+) denotes subjects who showed the amygdala low-frequency activity leading hippocampal gamma.

On the basis of the strong low-frequency phase coupling between the amygdala and hippocampus across all subjects, we then examined the directional influence of these responses with phase-amplitude coupling (PAC) across the two structures. To accomplish this, we again restricted our analysis to the most significant low-frequency phase coupling electrode pairs from the PLV analysis. Given that the electrodes with significant local PAC are more likely to engage in inter-regional coherence^25^,^26^, we first found that PAC within both the amygdala and the hippocampus was increased when viewing aversive stimuli compared with neutral stimuli (Supplementary Fig. 8). We then examined the directional PAC between the amygdala and hippocampus in the aversive condition compared with the neutral condition (Fig. 3a). We found that the HG amplitude in the hippocampus was phase locked to the amygdala low-frequency rhythms (all *P*<0.01, permutation test, Fig. 3a). The stronger coupling between the amygdala low-frequency phase and the hippocampus HG power was also observed in the theta trough-locked averaging of time–frequency plot in the aversive condition (*P*<0.05, Supplementary Fig. 9a), while this modulation was nearly absent when examining the reverse direction (for example, HG amplitude in the amygdala phase locked to the phase of hippocampal low-frequency activity. Fig. 3a and Supplementary Fig. 9b).

We also examined whether the PAC varied as a function of the time lag between low-frequency and HG signals. We posited that an amygdala to hippocampus directionality would result in a conduction delay^22^, which would translate to a relative phase shift between low-frequency and HG oscillations (Supplementary Fig. 12). Specifically, an earlier phase of amygdala low-frequency oscillations entraining hippocampal HG would result in the strongest PAC. We found that in the aversive condition, PAC between low frequency of the amygdala and HG of the hippocampus peaked around zero time lag (−13.15±2.92 ms versus hippocampus to amygdala directionality 13.52±22.83 ms), with 7 out of 9 subjects demonstrating that the amygdala low-frequency was leading the hippocampal HG (Fig. 3b, denoted by+near the red line, Pearson's *χ*^2^-test=6.73, *P*=0.035). In contrast, PAC between amygdala HG and hippocampus low-frequency was lower and failed to demonstrate a consistent peak at any time lag.

Since spectrally broad transients such as evoked activity can produce spurious PAC results^27^, we examined the spectral specificity of the modulated HG activity from the PAC results. To identify the rhythmic low-frequency fluctuation of the higher-frequency power time series, we determined the centre frequency of the HG signal from the PAC and filtered the raw signal within the HG band to extract the analytic amplitude of the signal (that is, envelope), which was then subjected to the PSD analysis. All subjects showed individual narrow-band low-frequency (4–12 Hz) peaks in the gamma envelope, thus supporting oscillatory properties of a separate low-frequency modulating signal in the HG band (Supplementary Fig. 10). To examine potential influence of eye movements on the PAC results, we ran an independent component analysis (ICA) on the EOG combined with white matter referenced amygdala and hippocampal activity in one participant (subject 9)^28^. Components composed of EOG activity (three components, based on mixing weights, Supplementary Fig. 4f) were removed from the raw intracranial data and all analyses were re-run with this ‘cleaned' data. The observed PAC effect remained significant after ICA correction (Supplementary Fig. 11). Further, there was no significant PAC between EOG channels and the amygdala as well as the hippocampus (Supplementary Fig. 5c,d). These findings indicate that eye movement contamination did not contribute to the original results.

### Granger causality and phase slope index

**Figure 4:**
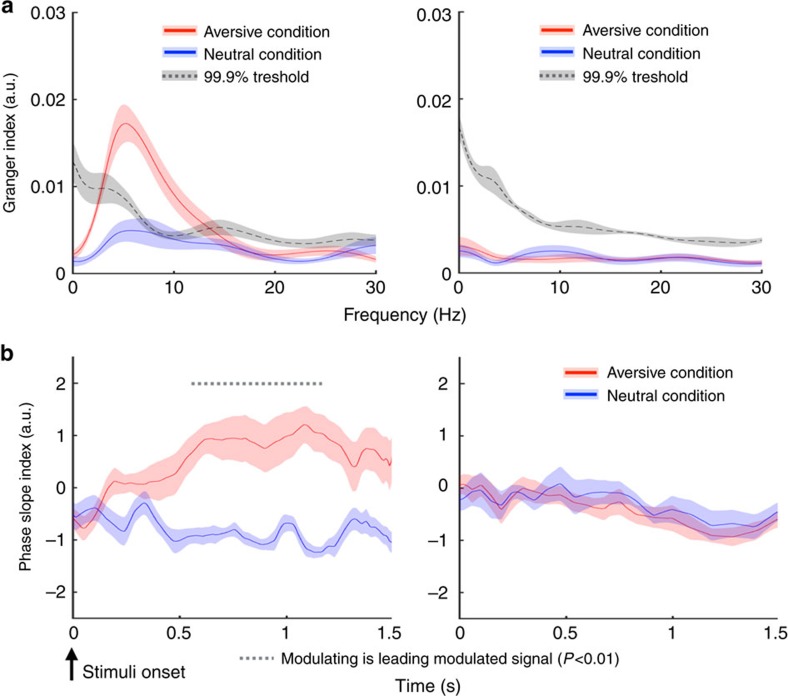
Granger causality analyses and PSI. (**a**) Granger causality analyses demonstrated consistently stronger influence for the amygdala-to-hippocampus direction (top left) than for the hippocampus-to-amygdala direction (top right) when contrasting the aversive to the neutral condition. The red and blue solid lines represent the real data from aversive and neutral conditions±s.e.m., respectively. Dotted lines denote 99% confidence intervals for the null distribution. (**b**) PSI between the aversive and the neutral conditions calculated point-by-point across time using the low-frequency signal from the modulating channel (coloured in red) and high gamma signal from the modulated channel (coloured in blue). Dotted lines above the graph denote significant differences between the two signals (all *P*<0.01, permutation test), showing that low-frequency activity from amygdala precedes hippocampus gamma for the majority of the stimuli duration (bottom left). The PSI did not show a directional influence from the hippocampus to amygdala (bottom right).

To further examine the directionality of the amygdala-hippocampal circuit, we utilized two complementary measures that rely on frequency and phase respectively: spectral Granger causality and phase slope index (PSI)^29^,^30^. The Granger causality measure quantifies the strength of directional influences between LFPs in the frequency domain by testing whether the LFP from one structure (for example, hippocampus) can be better predicted by incorporating information from the LFP from the other structure (for example, amygdala) and vice versa. PSI quantifies phase difference as a function of frequency, with a positive phase slope indicating that the signal from the first structure is leading the signal from the second structure. In low-frequency bands, we found significant Granger causal influence from the amygdala to hippocampus but not in the reciprocal direction (all *P*<0.01, permutation test, Fig. 4a and Supplementary Fig. 13a) only for the aversive condition. Significant differences between the aversive compared with the neutral condition were evident in eight out of nine subjects (Supplementary Fig. 14). The PSI analysis showed positive phase slopes from the amygdala to the hippocampus for the aversive compared with the neutral condition that remained significant between 0.58 and 1.16 s after stimulus onset (Fig. 4b and Supplementary Fig. 13b). In the reverse direction, the PSI did not show a positive value and there were no differences between the two conditions. These findings provide converging evidence of the amygdala to hippocampus directionality during processing of motivationally salient information.

## Discussion

Here, we demonstrate that processing of motivationally salient stimuli depends on coordinated neural oscillations between the amygdala and hippocampus, two critical nodes in the emotion processing circuit. Prior works have suggested that the amygdala rapidly detects salient stimuli, whereas the hippocampus engages in contextual and mnemonic processing^7^,^31^; however, the nature of their interaction and timing remains unclear. The current findings show that the interaction between the two structures is mediated by low-frequency phase coherence and that this relationship is directional, with amygdala low-frequency oscillations entraining hippocampal gamma. Our results are robust across individual subjects and provide a mechanism by which the amygdala influences hippocampal activity during recognition and emotional memory of salient information. We were able to disentangle the temporal dynamics of the interaction between the amygdala and hippocampus during processing of aversive stimuli by demonstrating an earlier post-stimulus onset of HG activity in the amygdala (as early as 120 ms post-stimulus) relative to the hippocampus (∼240 ms post-stimulus). Overall, these findings showed that salient stimuli are processed with distinct temporal windows in the amygdala and hippocampus. The early amygdala activity observed in this study may reflect the fast automatic detection of motivational salience of information, while the later hippocampal onset may indicate formation or reactivation of emotional memory^7^,^31^.

Previous research in affective processing using intracranial electroencephalogram (EEG) recordings in humans has focused on either the amygdala or hippocampus, but the oscillatory mechanism mediating communication across the two structures has remained unclear. Using retrograde tracing techniques in monkeys' hippocampal formation, Amaral and Cowan demonstrated that labelled neurons were found predominately in the anterior amygdaloid area, basolateral nucleus and periamygdaloid cortex^32^. The perirhinal and parahippocampal cortices have connections with the basolateral and accessory nuclei^33^. Although the primate tracing study could not provide anatomical specificity at the level of hippocampal subfields, rodent studies have shown that the ventral CA1 region and, to a lesser extent, CA3 receive the most robust amygdalar inputs^34^. Our findings are consistent with these known anatomical connections.

We further demonstrate that amygdala-hippocampal interactions were predominantly mediated through low-frequency coherence. Specifically, the PLV between the amygdala and hippocampus was enhanced when participants viewed fearful faces compared with landscapes. Given the long temporal window afforded by low-frequency rhythms, phase–phase coupling between different brain regions has been extensively studied in animal emotional research to understand regulation of inter-regional communication. In rodent models, the degree of fearful memory retrieval^35^,^36^ and its long-term consolidation^37^ are directly related to the degree of theta synchrony across the amygdala and hippocampus, implicating synchronous low-frequency oscillations as the neural correlate of fearful memory in the medial temporal network. Our study utilizing human intracranial EEG recording and precise medial temporal lobe subfield segmentation provides high-resolution temporo-spatial evidence for a similar oscillatory amygdala-hippocampal network in human salience processing and converges with the extant animal literature.

The directional influence of the amygdala onto hippocampus in anxiety and fearful emotional responses is increasingly recognized in animal models. Optogenetic manipulation of this circuit has demonstrated that selective activation of BLA terminals within the ventral hippocampus increases anxiety-related behaviour and reduces social interactions^10^. During contextual fear learning, enhancement of GABA neurotransmission in the BLA promotes hippocampal dendritic spine remodelling and fear retention, while blockade of GABA sites in BLA ameliorates these structural and behavioural effects^12^. In response to predatory threats, hippocampal place cells showed unstable firing patterns and increased theta power, an effect that was prevented by lesioning of the amygdala^11^. Thus, the modulatory effects of the amygdala on the hippocampus for processing of fearful stimuli are supported by animal studies demonstrating downstream alterations in neurotransmission, structure and electrophysiology. Our results provide confirmation of this directional modulation in humans and provide evidence that this modulation is controlled by neural oscillations. Specifically, amygdala low-frequency activity modulates hippocampal HG via amygdala-hippocampal theta/alpha synchrony during detection of motivationally salient information, providing a mechanism for the computation of local and long-range communication. These findings have implications for understanding deficits in the processing of salient environmental events in neuropsychiatric disorders with altered oscillatory control^38^.

There are several limitations in the current study. First, the two stimulus sets (fearful faces and landscapes) are inherently different in their properties. Thus, although we performed several analyses to show that evoked broadband activity did not influence the obtained results, potential contributions of evoked response could not be completely excluded. Second, the stimuli lacked neutral faces to contrast fearful faces and thus the differences between fearful faces and landscapes could be attributed to fearful content, emotion, facial expression, faces or other aversive stimuli. While our stimuli do not specifically address processing of fearful faces, contrasting fearful face movie clips with landscapes allows us to probe oscillatory mechanisms underlying processing of motivationally salient information. In accord with the recent literature highlighting a broader function of the amygdala, including processing of valence, emotion and value, we believe that our findings would generalize to other stimuli with social or survival significance^39^.

## Methods

### Participants

Data were obtained from nine patients (four female, five male, age 24–58, Supplementary Table 1) who had stereotactically implanted intracranial depth electrodes (Integra or Ad-Tech, 5 mm inter-electrode spacing) placed at the University of California, Irvine, Medical Center to localize the seizure onset zone for possible surgical resection. The institutional review boards of University of California at Berkeley and at Irvine approved the research, and written informed consent was obtained from each subject before testing. Electrode placement was exclusively guided by clinical needs, and patient selection was solely based on MRI confirmed depth electrode placement in the amygdala and hippocampus. Recordings were conducted from four patients (subjects 1–4) with depth electrodes localized ipsilateral and five patients (subjects 5–9) contralateral to or outside of seizure onset zone. There were no seizures recorded in any of the epochs, and any epochs with interictal epileptiform activity were removed from analysis. Comparable high gamma, PLV and PAC results were observed in all nine subjects, and there were no differences in the magnitude of effects between recordings from electrodes ipsilateral and contralateral to the seizure focus.

### Depth electrode localization

*MRI scans*. Electrodes were localized in each participant using co-registered pre-implantation and post-implantation structural T1-weighted MRI scans. The pre-implantation scans were all 1 mm isotropic. The post-implantation scans were either 1 mm isotropic (subjects 1, 5, 6, 8 and 9) or 0.75 × 0.75 × 7 mm (subjects 2–4 and 7). For each participant, the post-implantation scan was registered to the pre-implantation scan using a six-parameter rigid body transformation (three rotations and three translations in *x*–*z* directions), implemented in Advanced Normalization Tools (ANTs http://stnava.github.io/ANTs/)^40^.

*Anatomical masking*. For determining exact electrode locations, we used a high-resolution anatomical template (0.55 mm) developed in our laboratory with manual tracings of hippocampal subfields and amygdala nuclei. This template has been used in past studies^41^,^42^,^43^. Regions of interest (ROIs) in the medial temporal lobe included the DG/CA3, CA1, subiculum, entorhinal cortex, perirhinal cortex, parahippocampal cortex, BLA, central nucleus of the amygdala (CeA), and the cortical nuclei of the amygdala (CORT)^44^,^45^. Segmentations for hippocampal subfields followed our previously published protocols^46^. Briefly, the segmentation included DG/CA3, CA1 and subiculum, and procedures followed closely the atlas of Duvernoy^44^, in which the subfields are defined along the anterior-posterior axis of the hippocampus. Amygdala segmentation procedures were based on Entis *et al*.^45^, but were modified to define three regions: BLA, CeA and CORT. To label these sub-regions of the amygdala, three key points were identified: (1) the medial tip of the alveus (up to the optic tract); (2) the most lateral point of the entorhinal sulcus; and (3) bottom of the circular sulcus. These three points are easily observable and provide a reliable landmarking system for segmenting the amygdala sub-regions. After identifying these points, lines were drawn to connect the three points to each other, creating four quadrants (basomedial and basolateral were combined to form the basolateral complex).

*Labelling individual participant scans*. The labelled high-resolution anatomical template (resampled to 1 mm isotropic) was aligned to each individual's pre-implantation scan using ANTs Symmetric Normalization^40^, such that the labels could be applied to each participant's MRI. This allowed for visualization and accurate identification of electrode locations using anatomical labels in each participant's space. Each electrode location was determined by selecting the centre of the electrode artefact and identifying the region of interest that encompassed the centre. Cases where electrodes were on the border between ROI's or between grey matter and white matter were noted as such.

### Behavioural task

Participants watched silent movie clips on a laptop computer placed on the service tray at a comfortable distance in front of them. The total length of the task was about 7 min, consisting of alternating 9 blocks of landscapes and eight blocks of fearful faces (∼24 s/block, see Fig. 1a). During the experiment, participants viewed 70 landscapes and 71 fearful face clips (clip duration: 2.8±1.3 s; mean±s.d.). Within each block, movie clips were shown continuously without breaks. This paradigm was used in previous fMRI studies and demonstrated reliable amygdala activation^47^,^48^.

### Data collection and preprocessing

Intracranial EEG data were acquired using a Nihon Kohden recording system (256 channel amplifier, model JE120A), analogue-filtered above 0.01 Hz and digitally sampled at 5,000 Hz. All the data were analysed in MATLAB combined with open source toolboxes and custom scripts. After acquisition, neuronal recordings were band-pass filtered from 0.1 to 350 Hz using a zero phase delay finite impulse response (FIR) filter with Hamming window and then down-sampled to 2,000 Hz for subsequent analyses. A regression method^49^ was used to filter 60 Hz noise and its harmonics. All hippocampal and amygdala electrodes were re-referenced to the nearest electrode located in white matter. A neurologist with subspecialty training in epilepsy (J.J.L.) manually inspected all EEG signals to identify and remove all data with interictal epileptiform discharges as well as excessive noise, including broadband electromagnetic noise from hospital equipment. To avoid potentially biasing the results, the neurologist was blinded to the location and behavioural task (for example, aversive versus neutral condition) associated with EEG signals and the artefact rejection was performed before additional data processing.

### Electro-oculogram and perisaccadic high gamma activity

To investigate the possibility of eye-movement-related contamination of our results, one subject (S9) was recorded along with a free head fixation eye tracker (SensoMotoric Instruments, Inc. RED 250 mobile) and EOG recordings. The eye tracker, connected to the laptop with Universal Serial Bus (USB), was placed at the inner edge of the presentation laptop. Before the task, the eye tracker was calibrated using the iView RED-m software. Timing between the eye tracker recording and the video clips was synchronized through an E-Prime script. After averaging the velocity of the eyes in the *X* and *Y* dimensions from the eye tracker data across clips, two-sample *t*-tests were performed for each data point to test for significant differences in patterns of eye movements between conditions that could affect the results. In addition to the eye movement analysis, we estimated the time course of high gamma activity (HG) in the vertical and horizontal EOG. Again, two-sample *t*-tests were performed for each data point to test for significant differences between conditions. As a third test to investigate the contribution of ocular activity to our findings, we used ICA (Bell-Sejnowski ICA algorithm)^28^, implemented in the EEGLAB toolbox for Matlab on the EOG combined with white matter re-referenced amygdala and hippocampal activity to compute the weights of contributions from each channel of data to each independent component. Any components composed of mostly EOG activity were removed from the data by zeroing those components and re-projecting the remaining components into the channel space. All analyses were then re-run with this ‘corrected' data.

Saccades were detected through a velocity-based algorithm^6^. The velocity of eye movement was estimated as the first derivative of the Euclidean distance between eye positions at successive sample points. A threshold was set at the 99th percentile of velocities and saccades were marked at the time points of the peak velocities that surpassed this threshold, with a minimum spacing of 200 milliseconds between saccade events. High gamma (70–180 Hz) activity in hippocampal and amygdala contacts was then epoched based on these events. After baseline correction (−200 ms to 0 ms), two-sample *t*-tests were performed for each data point to determine significant differences between conditions. To further investigate the possibility that PAC analyses could have been influenced by EOG activity, we calculated the PAC using the gamma-range component of the EOG signal and low-frequency phase from the hippocampus and amygdala (the same analysis as described in the Methods ‘Phase-amplitude coupling' section). We observed no significant PAC pattern between the amygdala or hippocampus and the EOG HG signal, which indicates that there was no appreciable influence from potential eye movement artifacts on the PAC analysis.

### Power spectral density (PSD) and subject-specific low-frequency band

PSD was estimated using Welch's method^50^ (pwelch.m in Signal Processing Toolbox from MATLAB) wherein the PSD was estimated for each subject separately using 1 s time windows with a 50% overlap. The slope of the power spectrum was estimated using the linear regression approach in a semi-log space, where the power *P* at each discrete frequency *f* was estimated from the frequency itself using the following formula:


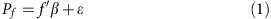


where 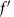 is a two column matrix composed of the discrete frequency bands of interest and a column of ones; *β* is the regression coefficient (the slope of the model), and *ɛ* is the error term. In intracranial power spectra, *β* is typically negative, which is important given that task-related increases in neural activity result in a broadband upward shift within the high gamma range^51^. To characterize spectral dependence between the amygdala and hippocampus in a narrow band, we selected the subject-specific low-frequency band based on the PSD plots. Specifically, the value of exponent *χ* in the power-law relation 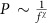 was obtained by fitting a straight line to the experimentally measured PSD. Then the distances between the PSD and the fitting curve were calculated at each frequency point. The subject-specific band was defined as the farthest points from the fitting curve within the low-frequency range with a bandwidth of 4 Hz.

### Frequency decomposition

Data from the amygdala and hippocampus were first filtered into subject-specific low-frequency bands (Amygdala: *x*_Amy*θ*_=6.0±0.49 Hz; hippocampus: *x*_Hipp*θ*_=6.5±0.16 Hz; mean±s.e.m.) and high gamma (*x*_*γ*_, 70–180 Hz) using a two-way, zero-phase lag, least-squares FIR filter to prevent phase distortion (eegfilt.m function in EEGLAB toolbox^52^). The length of the filter in points was determined by the specific frequency, cycle number (usually 4–5 cycles) and sampling rate. The centre frequency of each frequency bin is spaced apart by 0.1 of the lower-frequency band and the bandwidth varied by multiplying the fractional bandwidth index 0.3 with the centre frequency. For example, for the frequency bin centred at 10 Hz, the next frequency bin is 10 × 0.1+10=11 Hz and the bandwidth is 10 × 0.3 × 2=6 Hz. Since we choose adaptive rather than fixed bandwidths, the bandwidths within low-frequency range were sufficiently narrow to define a meaningful phase while the bandwidths within higher-frequency range were broad enough to fit the sidebands caused by the assumed modulating lower-frequency band^27^. We then applied the Hilbert transform (hilbert.m function in Signal Processing Toolbox from MATLAB) to estimate the amplitude (*a*_*x*_[*n*]) and phase (*θ*_*x*_[*n*]) for both bands, which yields a complex time series:


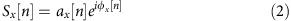


where *a*_*x*_[*n*] represents the instantaneous analytic amplitude and *φ*_*x*_[*n*] is the instantaneous phase.

### Event-related potentials

The ERPs were calculated individually for both the amygdala and hippocampus separately. The LFP signals were first low-pass filtered at 30 Hz using the same filter parameters as described in Methods (‘Frequency decomposition' section). Then the filtered signals were segmented into epochs, and the ERPs were calculated by averaging across these epochs within each condition. After baseline correction by subtracting the mean baseline (−500 ms to 0 ms) value from all data points, two-sample *t*-tests were performed for each data point to determine significant differences between conditions.

### High gamma activity

To investigate the time course of HG activity within the amygdala and hippocampus, we extracted the high-frequency banded signal using the frequency decomposition and averaged the results within each region. Specifically, the HG amplitude data were divided into epochs from −0.5 to 1.5 s from the start of each movie clip. Data within each trial were normalized by converting each data point into a *z*-score relative to the entire trial time-series. The computed *z*-scores were then baseline corrected by subtracting an average of pre-stimulus baseline data points (a 500 ms time period taken from the beginning of the session, when the blank screen was presented, see Fig. 1a). This allows us to eliminate the positive skew typically observed for HG amplitude values^53^. We then statistically compared the event-related HG amplitude changes between the landscape and the fearful face conditions using a cluster-based permutation test, computing statistics at the cluster level and correcting for multiple comparisons^54^. First, all the trials from the two experimental conditions were randomly shuffled, and the means of each condition were subtracted 1,000 times to create a null distribution of differences between conditions. *T*-scores were then computed for each null difference time series by comparing it to the entire distribution of null difference time series at every time point. Then, all *t*-scores corresponding to uncorrected *P* values of 0.05 or less were formed into clusters with any neighbouring such *t*-scores. The sum of the *t*-scores in each cluster is the ‘mass' of that cluster and the most extreme cluster mass in each of the 1,000 sets of tests was recorded and used to estimate the distribution of the null hypothesis. Finally, clusters were obtained from the true data, and the percentage of null cluster masses greater than each true data cluster mass was taken as the corrected *P* value for that cluster. Onsets and offsets of the HG activity for each condition were computed by taking the first and last time sample that passed a significance level of *P*<0.05. Time-to-peak was defined for fearful face and landscape conditions as the latency of the maximum amplitude in the HG range across all trials within each condition during the range of time where a significant difference between the two conditions was found.

### Phase locking value (PLV)

To quantify the inter-electrode low-frequency phase coupling, phase differences were calculated for each electrode pair (*i*, *j*) using the phase time series *φ*_*x*_[*n*] obtained from equation (2). The frequency range covers the low-frequency peaks from both the amygdala and hippocampus (identified in PSD) with a bandwidth of 4 Hz. The phase difference between the two time courses *φ*_*θij*_[*n*] indexes the coherence between each electrode pair and is expressed as the PLV index (equation (3)). PLVs range between 0 and 1, with values approaching 1 if the phase differences between the electrodes vary little across time.


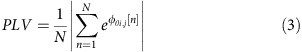


The filtered data were segmented into one-second windows and then separated according to task condition before computing a PLV for each electrode pair and condition. Relative increases (or decreases) in PLV in response to fearful faces versus landscape scenes were assessed by subtracting the two PLVs for each electrode pair. To test the significance of differences between two conditions and, at the same time, maximally eliminate the influence of ERPs, we did a two-step approach as described below. We first permuted trials and computed the 99th percentile threshold within each condition. Only the electrode pairs whose PLVs rose above the threshold for both conditions were selected for calculating the PLV difference. Then the statistical significance for each electrode pair was estimated using a cluster-based permutation test, in which a null distribution was created by randomly assigning trials (i.e., one-second windows) into two conditions, computing the relative PLV differences between conditions, and repeating this procedure 1,000 times. The observed data were then compared with this null distribution to estimate a *P* value. The results were depicted as hive plots to visualize the magnitude of the PLV and the associated *P* values among pairs of electrodes^55^.

To test the regional specificity of this phase coupling effect, an analysis of variance with pairwise comparisons was conducted to test differences between hippocampal sub-regions using the R statistic toolbox. First, the Shapiro–Wilk test (function Shapiro.test) was conducted to test whether the data were approximately normally distributed. Second, the homogeneity of variances was tested using Bartlett's test (function Bartlett.test). Then the pairwise comparison was computed using the function pairwise *t*-test. In addition, to further address the continuous consistency of the phase relationship between the amygdala and hippocampus, the PLV spectra were created as a function of frequency (1–30 Hz) for all BLA and hippocampal electrode pairs averaged across all subjects for each condition and the difference was calculated by subtracting neutral from aversive condition and *z*-score normalized. A fifth-order spline interpolation was implemented (spapi.m in Curve fitting toolbox from MATLAB) to smooth the curve of PLV difference spectra.

### Phase-amplitude coupling

On the basis of the previous phase coupling analysis, we analysed the PAC between BLA and hippocampus electrode pairs that exhibited the most significant PLV in each patient. To extract the directionality information from these electrode pairs, the relationship between lower-frequency (1–30 Hz; delta, theta, alpha, beta) phase from electrode *i* and higher-frequency (30–250 Hz; gamma) amplitude from electrode *j* was examined individually for each condition by calculating the circular linear correlation between the instantaneous phase of low-frequency and the instantaneous phase of high frequency^56^.


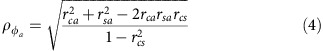


Where *r*_*ca*_=*c*(cos*φ*_*θi*_[*n*], *a*_*γj*_[*n*]), *r*_*sa*_=*c*(sin*φ*_*θi*_[*n*], *a*_*γj*_[*n*]) and *r*_*cs*_=*c*(sin*φ*_*θj*_[*n*], cos*φ*_*γj*_[*n*]) with *c*(*x*, *y*) equal to the Pearson correlation between *x* and *y*, *φ*[*n*] equals to the instantaneous phase from the modulating signal, and *a*[*n*] equals to the instantaneous analytic amplitude from the modulated signal. To compare the significance of the difference between correlation coefficients *ρ*_1_ and *ρ*_2_, we applied Fisher's *z*-transform to normalize correlation coefficients such that 
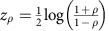
, and calculated the difference Δ*ρ*_*z*_=*z*(*ρ*_1_)−*z*(*ρ*_2_) (ref. 57). The permutation test described for the previous analyses was also used here to create a null distribution of PAC differences between two conditions for each electrode pair. Then the *z*-PAC was defined as the *z*-score of the real difference in PAC between the two conditions based on the distribution of phase-amplitude coupling values obtained from these null distributions with the positive value indicating PAC increase in the aversive condition versus neutral condition.

### Phase-amplitude coupling with time lag

As the communication between the modulating and modulated signal builds, the carrier oscillation is likely to appear first in the modulating signal. In other words, immediately preceding the synchronization between two signals, the ‘driver' signals should be predictive of the ‘receiver' signal. To probe the direction of functional coupling and to further explore how the neuronal dynamics in low-frequency and gamma bands interact, we examined the PAC magnitude as a function of the time lag between modulating and modulated signals. A diagram of the data processing steps is shown in Supplementary Fig. 12. The cross-regional PAC was calculated using the same algorithm as outlined in the PAC analysis section but with different time lags (in 10 ms intervals for the total duration range of ±200 ms) by shifting one signal relative to the other one.

### Power spectral density (PSD) of the high gamma envelope

The goal of this step was to identify rhythmic fluctuations present in the higher-frequency power time series. We tested the assumption that if the power time series within the higher-frequency band are synchronized with a lower-frequency oscillation, the power time series will not be constant over time but instead will fluctuate at the specific lower oscillation frequency^58^. For example, if a signal banded at 80 Hz is synchronized with a 6 Hz oscillation, the power time series of the 80 Hz might itself oscillate at 6 Hz. To test this hypothesis, we first selected the subject-specific high-frequency band centred at the frequency with the strongest PAC and bandwidth of 40 Hz. Then, the PSDs of the selected high-frequency bands were calculated using the same methods as described in Methods (‘Power spectral density and subject-specific low-frequency band' section).

### Theta-trough locked averaging of time–frequency plot

A theta-trough locked time–frequency averaging plot for a single subject was created to demonstrate the oscillatory features of the underlying signal^5^. To create the tracing in the lower panels of Supplementary Fig. 9, the raw signal from the amygdala/hippocampus was first filtered within subject-specific low-frequency band (5.8–9.8 Hz for this subject) using the same FIR filter parameters as described in the Methods (‘Frequency decomposition' section). By applying the Hilbert transform, the phase at each time point was extracted from the filtered data within (−*π*, *π*], where *π* radians corresponded to a theta trough and 0 radians corresponded to a theta peak (cosine phase). Then, the signal was segmented into 1 s epochs centred at the theta troughs, which were identified as the local minima of the phase less than (−*π*+0.01) and the theta-trough locked ERP were generated by averaging across all epochs. For Supplementary Fig. 9 upper panel, a set of normalized instantaneous power time series from the hippocampus/amygdala was constructed, with centre frequencies ranging from 1 to 250 Hz. To facilitate comparisons between different frequency bands, the band-pass filtered signals within the specific frequency range were first normalized by subtracting the temporal mean and dividing by the temporal s.d. The normalized instantaneous power time series were generated by applying the Hilbert transform and taking the square of the extracted amplitude time series. These power series were then segmented into the epochs centred on the corresponding theta trough (Supplementary Fig. 9 lower panel), *P* values for each time point in each frequency band were calculated using the non-parametric permutation test described in Methods (‘High gamma activity' section).

### Phase slope index (PSI)

As an index of dominant unidirectional interaction^30^, PSI indicates the direction of coupling between two systems. Given a pre-specified bandwidth parameter, it reflects the change of phase difference between neighbouring frequency bins, weighted with the magnitude of the coherence. On the basis of the assumption that independent sources do not contribute to the imaginary part of the cross-spectrum, the PSI is defined as


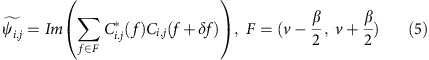


where 
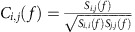
 is the complex coherence, 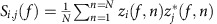 is the cross-spectrum between two time series, *ν* is the centre frequency of the targeted frequency range, and *Im* denotes the imaginary part. We use *β* as the bandwidth for which the phase slope is calculated and choose it to be eight times of the frequency resolution *δf*29. For example, when calculating directionality for a phase modulation centred at 8 Hz rhythm with a 1 Hz frequency resolution, a phase slope estimated between 4 and 12 Hz is reasonable (*β*=4 *Hz*). To understand interaction and directionality between the signals involved in cross frequency coupling, we segmented the phase of the modulating signal and the power envelope of the modulated signal into N epochs and used them as inputs to calculate the PSI for both aversive and neutral conditions. As the interactions between modulating and modulated signals require certain amount of time, and if the speed at which these waves travel is similar, the sign of the PSI informs about which signal is temporally leading the other one. In other words, when the phase differences between the ‘sender' and ‘recipient' signal increase with the corresponding frequencies, a positive slope of the phase spectrum is expected. By performing the PSI analysis with a sliding window of 100 ms, spaced at 25 ms (75% overlap), we were able to track the switching of the directionality between two signals at each time point. To assess the statistical significance of PSI, we applied the analogous non-parametric approach as describe above by randomly shuffling the trials within a condition. Then the PSI null distribution was created at each time point, and the 99.5th percentile threshold was defined for each condition separately.

### Granger causality

To further investigate interactions between brain regions, we computed spectral Granger causality. Granger causality represents how much introducing past measurements from a first time series can decrease the variance of the prediction error for a second time series at the current time point. Spectral Granger causality extends this analysis to the frequency domain^59^,^60^. The time-domain data was first low-pass filtered at 85 Hz and then down-sampled to 250 Hz before fitting to an autoregressive model and computing spectral Granger causality. The model order m was determined by the Akaike information criterion^61^ (using the Multivariate Granger Causality (MGVC) Matlab Toolbox), which is a tradeoff between sufficient spectral resolution and over-parameterization. Model orders were estimated for each patient and varied from 7 to 13. The Granger index was computed using the first 1,500 ms from each clip as trial realizations. To further address whether this directionality was task specific, the null hypothesis distributions of spectral Granger causality for each condition were created by randomly swapping clip segments between channels. The spectral Granger causality was considered significant if they exceeded the 99% confidence interval of this null hypothesis distribution. To further test whether the Granger causality is significantly different between conditions, the null difference time series were computed by subtracting the Granger time series from the aversive condition and the ones from the neutral condition, which were calculated by randomly flipping the segments between conditions. Then for each frequency point, there were two Granger values as the upper or lower range of the 99% confidence interval, where 98% of the null Granger difference time series were either less or greater than these values. It shows a greater Granger for the aversive condition compared with the neutral one if the real difference line is above the upper confidence interval while the neutral condition has a stronger Granger if the real difference line is below the lower confidence interval.

### Data availability

The data that support the findings of this study are available from the corresponding author upon request.

## Additional information

**How to cite this article:** Zheng, J. *et al*. Amygdala-hippocampal dynamics during salient information processing. *Nat. Commun.* **8,** 14413 doi: 10.1038/ncomms14413 (2017).

**Publisher's note:** Springer Nature remains neutral with regard to jurisdictional claims in published maps and institutional affiliations.

## Supplementary Material

Supplementary Information Supplementary Figures and Supplementary Table.
