# Supplementary material for: Amygdala-hippocampal dynamics during salient information processing
Source: Nat Commun. 2017 Feb 8;8:14413. doi: 10.1038/ncomms14413 (PMC5309795; doi:10.1038/ncomms14413)
Supplement: Supplementary Information — Supplementary Figures and Supplementary Table. [file ncomms14413-s1.pdf]

Supplementary Information

Supplementary Table 1: Patient information

Subject	Implantation Side	Age	Gender	MRI	Surgery	Side Analyzed
1	Right	24	Male	Right MTS	Right ATL with AH	Right
2	Left	25	Male	Normal	Left ATL with AH	Left
3	Bilateral	51	Male	Normal	Right ATL with AH	Right
4	Left	49	Female	Normal	Left ATL with AH	Left
5	Bilateral	33	Female	Right MTS	Right ATL with AH	Left*
6	Bilateral	32	Female	Right periventricular heterotopia	Right ATL with AH	Left*
7	Right	25	Male	Normal	Right ATL without removing Amygdala and Hippocampus	Right*
8	Bilateral	34	Male	Normal	Right ATL with AH	Left*
9	Bilateral	48	Female	Normal	Left ATL with AH	Right*

ATL anterior temporal lobectomy; AH = amygdalohippocampectomy

MTS = mesial temporal sclerosis

*= Contralateral or outside of the seizure onset zone

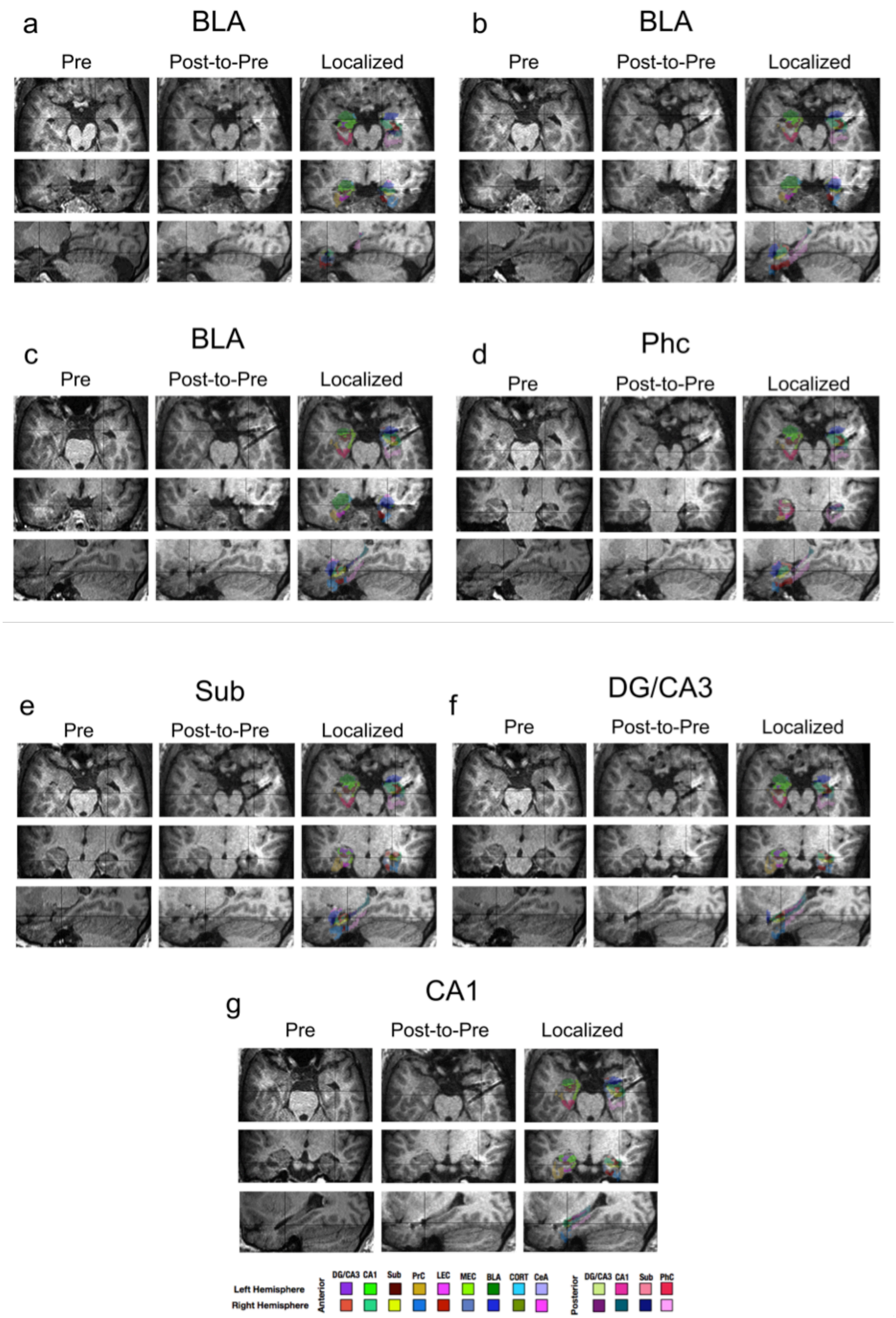
a
BLA
Pre
Post-to-Pre
Localized
b
BLA
Pre
Post-to-Pre
Localized
c
BLA
Pre
Post-to-Pre
Localized
d
Phc
Pre
Post-to-Pre
Localized
e
Sub
Pre
Post-to-Pre
Localized
f
DG/CA3
Pre
Post-to-Pre
Localized
g
CA1
Pre
Post-to-Pre
Localized
Left Hemisphere
Right Hemisphere
Anterior
DG/CA3
CA1
Sub
PrC
LEC
MEC
BLA
CORT
CeA
Posterior
DG/CA3
CA1
Sub
PhC

Supplementary Figure 1. Localization of depth electrodes. Pre-implantation MRI (1 mm isotropic) in a representative subject (left panel), post-implantation MRI aligned using rigid body alignment to the pre-implantation MRI (middle panel), and the translucent overlay of registered regions of interests (ROIs) overlaid on the post-to-pre aligned MRI (right panel) for the basolateral amygdala (BLA; a-c), parahippocampal cortex (Phc; d), subiculum (Sub; e), dentate gyrus/CA3 (DG/CA3; f), and CA1 (g). Cross hairs are centered on the electrode in each region.

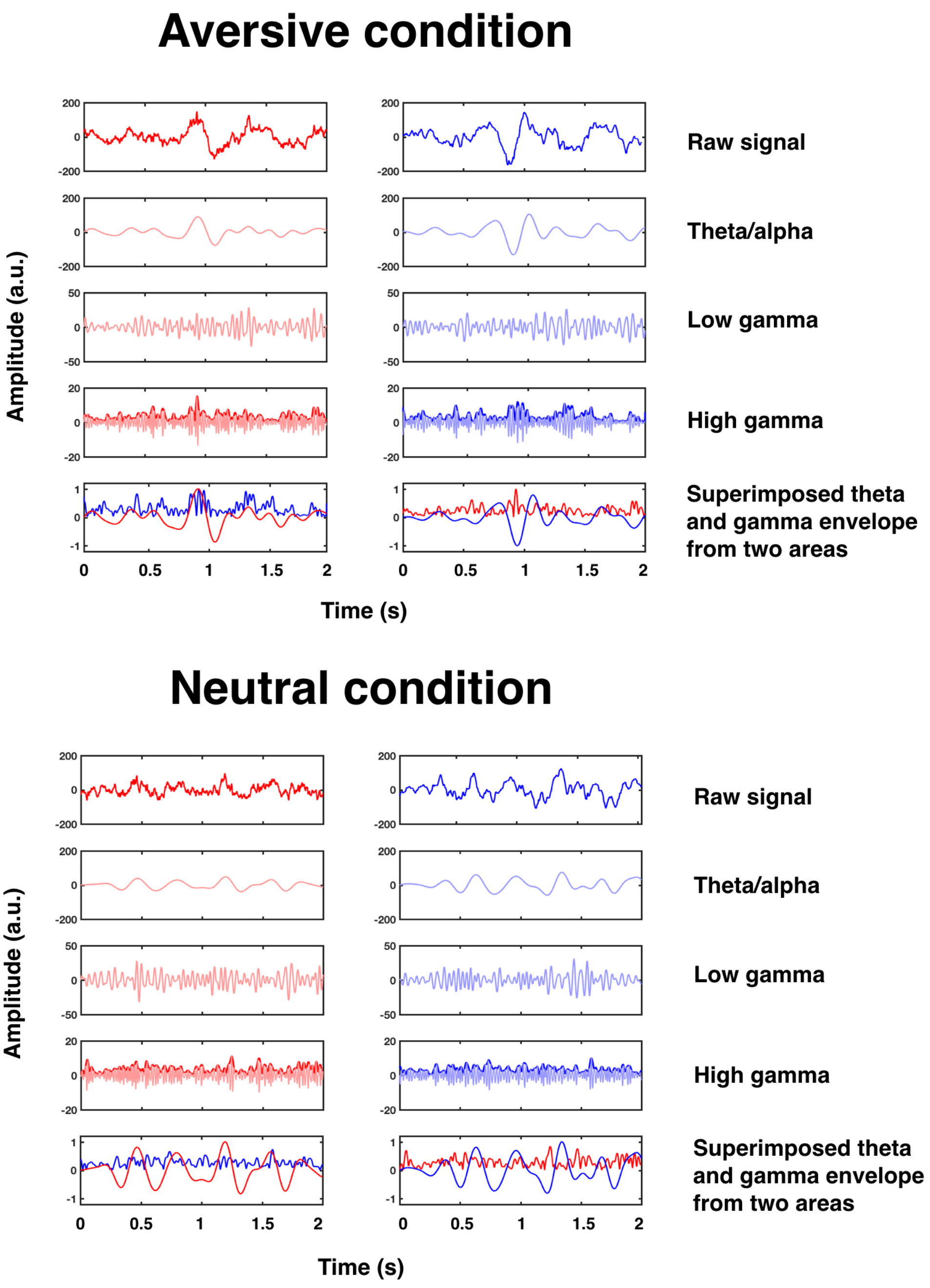

Supplementary Figure 2. **Raw traces of a single trial for the aversive and the neutral conditions in the amygdala and the hippocampus.** Traces from top to bottom show unfiltered raw, filtered theta/alpha (5-9 Hz), filtered low gamma (30-70 Hz), and filtered high gamma (70-180 Hz) signals, with the analytic amplitude envelope traced in bold. The lowest row in each panel shows the high gamma banded amplitude envelope from one region that is superimposed with the low frequency banded signal from the other region. A stronger synchronization between amygdala theta and hippocampus gamma is evident in the aversive condition compared to the neutral condition. Red = signals from the amygdala; blue = signals from the hippocampus.

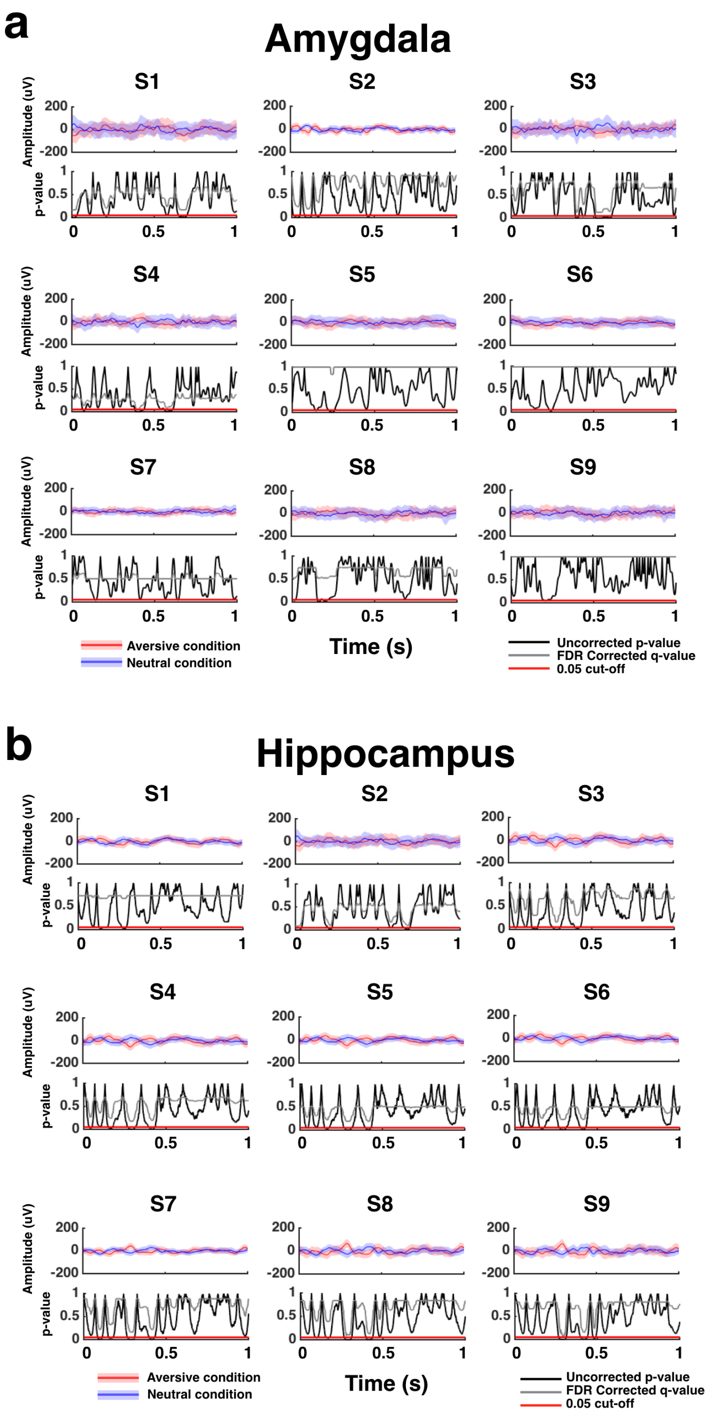

Supplementary Figure 3. Movie clip-based event related potentials (ERPs) in each subject for (a) the amygdala and (b) the hippocampus. Comparisons of ERPs between the aversive (red traces) and neutral (blue traces) condition (top colored traces) and point by point *t*-tests (bottom traces). Significant group differences in ERP amplitudes with uncorrected $p < 0.05$ threshold were found for a small number of time points (amygdala = 6.5% and hippocampus = 8.3%). No data points survived correction for multiple comparisons. Shaded regions denote s.e.m. across trials.

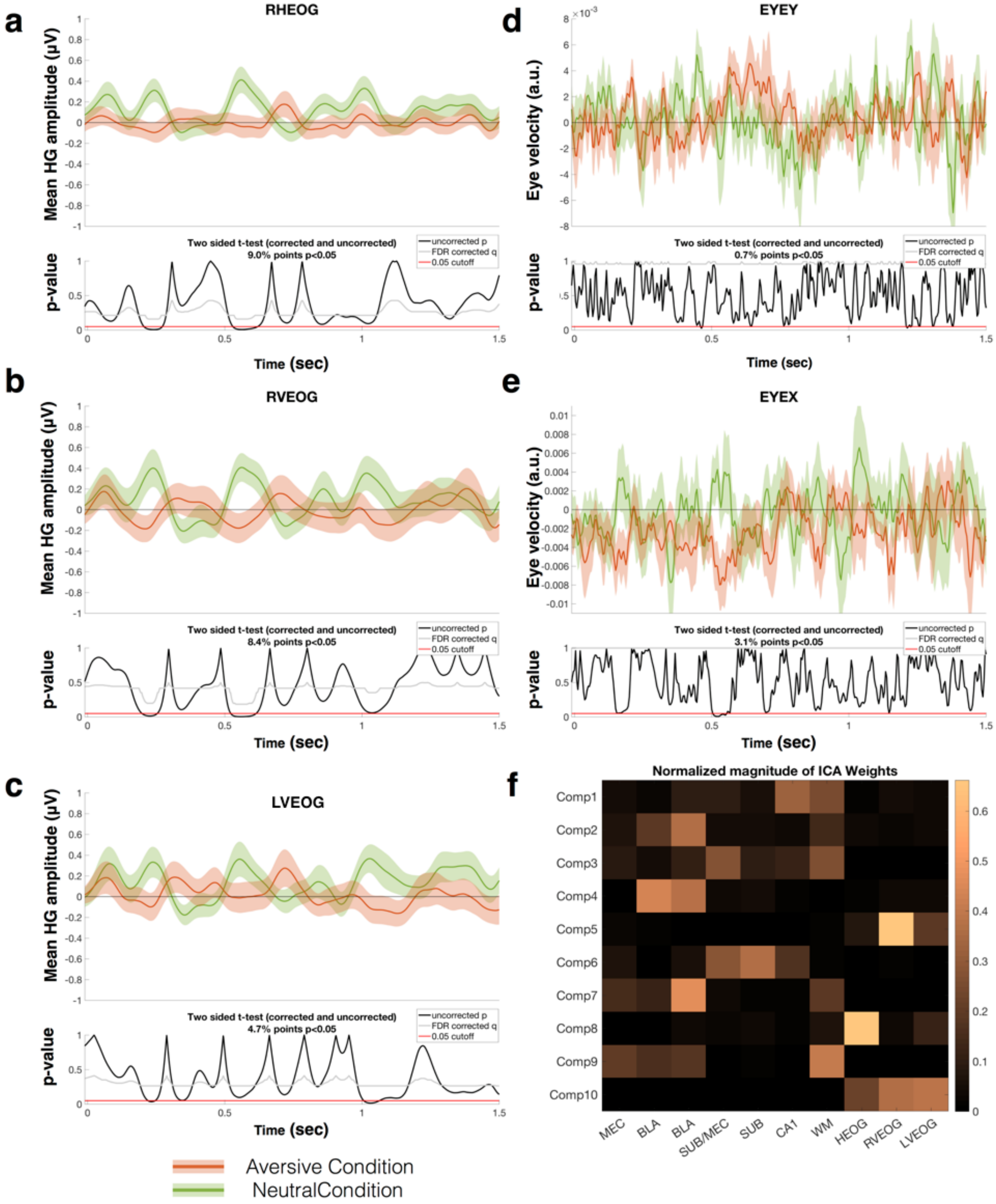
a
RHEOG
Mean HG amplitude (μV)
Two sided t-test (corrected and uncorrected)
9.0% points p<0.05
uncorrected p
FDR corrected q
0.05 cutoff
p-value
Time (sec)
b
RVEOG
Mean HG amplitude (μV)
Two sided t-test (corrected and uncorrected)
8.4% points p<0.05
p-value
Time (sec)
c
LVEOG
Mean HG amplitude (μV)
Two sided t-test (corrected and uncorrected)
4.7% points p<0.05
p-value
Time (sec)
d
EYEY
Eye velocity (a.u.)
Two sided t-test (corrected and uncorrected)
0.7% points p<0.05
p-value
Time (sec)
e
EYEX
Eye velocity (a.u.)
Two sided t-test (corrected and uncorrected)
3.1% points p<0.05
p-value
Time (sec)
f
Normalized magnitude of ICA Weights
Comp1
Comp2
Comp3
Comp4
Comp5
Comp6
Comp7
Comp8
Comp9
Comp10
MEC
BLA
BLA
SUB/MEC
SUB
CA1
WM
HEOG
RVEOG
LVEOG
Aversive Condition
NeutralCondition

Supplementary Figure 4. Eye movement related responses and independent component analysis for Subject 9. Comparisons of estimated high gamma time courses from the electrooculogram (EOG) activity between the aversive (fearful face) and neutral (landscape) condition (top colored traces) and point-by-point t-tests (bottom traces) for (a) the right horizontal EOG (RHEOG), (b) right vertical EOG (RVEOG) and (c) left horizontal EOG (LVEOG). A small number of data points were found to be significant at the uncorrected $p < 0.05$ threshold (RHEOG = 9.0%, RVEOG = 8.4%, LVEOG = 4.7%), and multiple comparison correction eliminated any significant differences. Ocular velocity in (d) the X dimension and (e) the Y dimension recorded from the eye tracker averaged across clips and compared between the two conditions (top colored traces) and the point-by-point *t*-tests (bottom traces). A small number of data points were found to be significant at the uncorrected $p < 0.05$ threshold (X= 0.7% and Y = 3.1%), and no significant differences were present after multiple comparison correction. (f) Independent component analysis (ICA) of EOG combined with white matter referenced amygdala and hippocampal activity. ICA weights were normalized by taking the magnitude (absolute value) of all weights and then dividing all weights across each row (component) by the sum of all weight magnitudes in each row. This effectively shows the relative contributions of channels to each component. EOG signals contributed to components 5, 8, and 10, which had relatively small contributions from amygdala and hippocampal electrodes. The sub-regions used for the eye movement analyses were the same as in Fig 2, subject 9. HG = high gamma; a.u.=arbitrary units

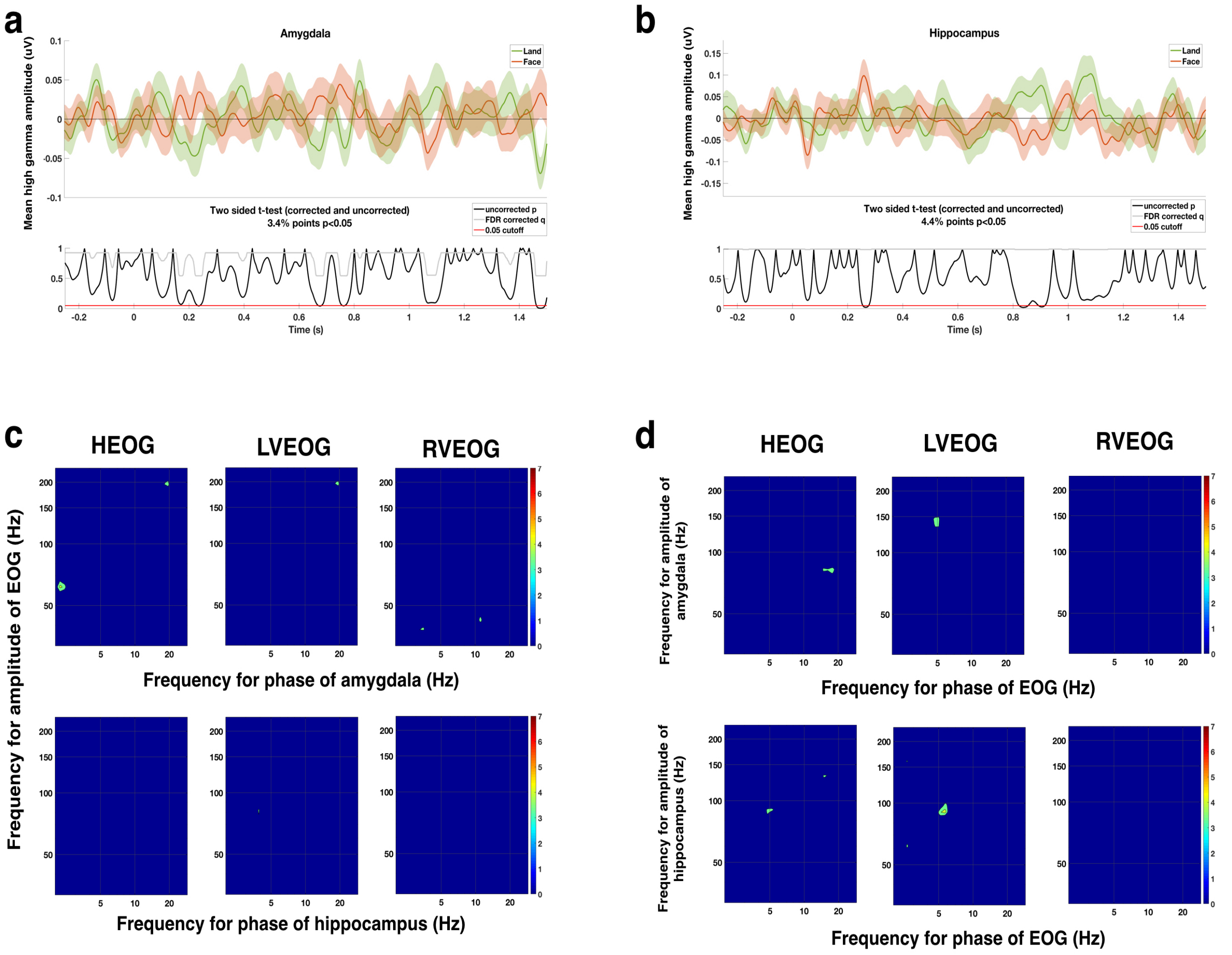

Supplementary Figure 5. Perisaccadic high gamma activity and electrooculogram (EOG) derived phase amplitude coupling for subject 9. Comparisons of estimated high gamma time courses from (a) the amygdala and (b) the hippocampus between the aversive (fearful face) and neutral (landscape) condition. A small number of data points were found to be significant at the uncorrected $p < 0.05$ threshold (amygdala = 3.4%, hippocampus = 4.4%), and multiple comparison correction eliminated any significant differences. No significant phase amplitude coupling pattern was observed between the EOG activity and signals from the amygdala and hippocampus (c, d).

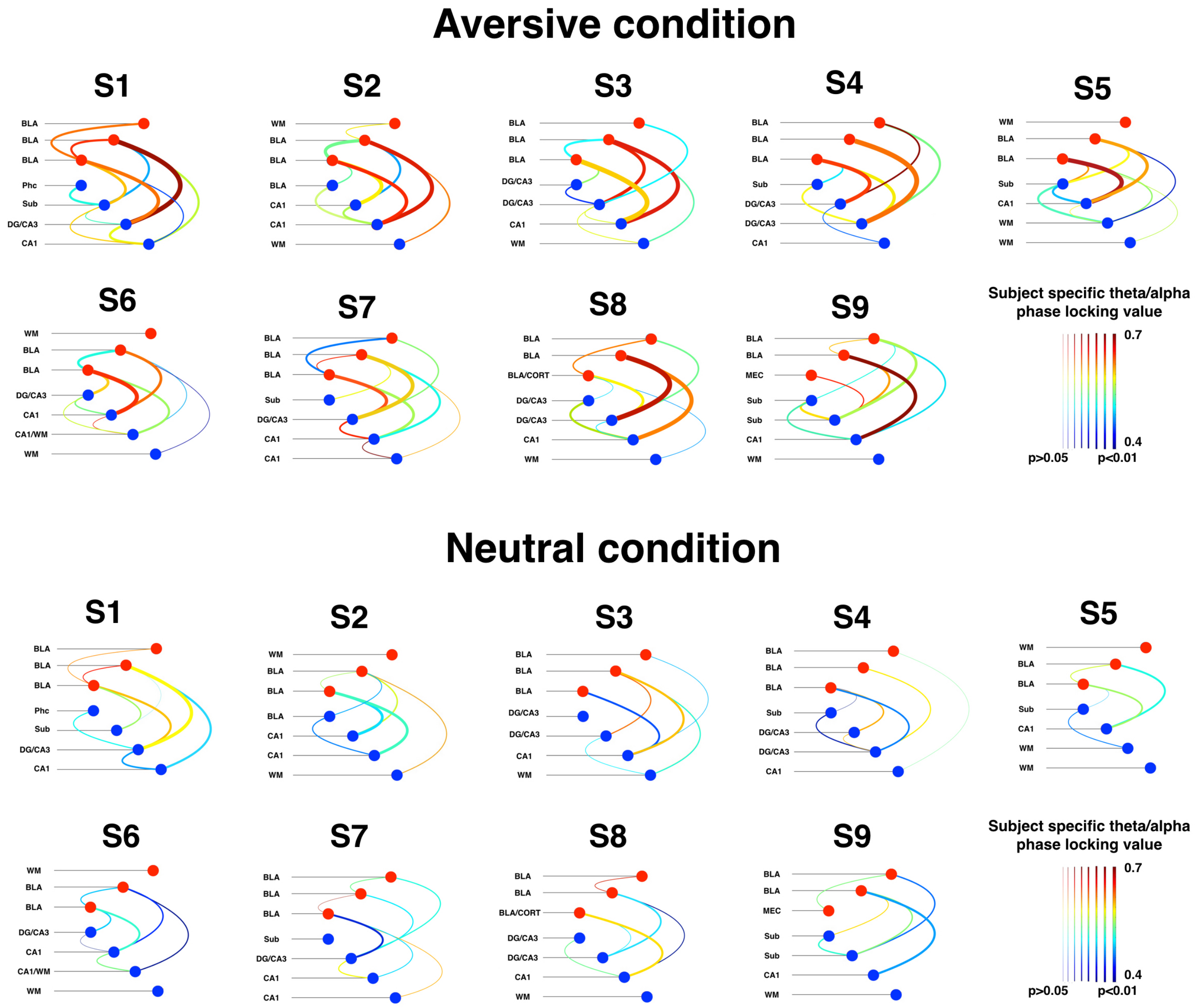

Supplementary Figure 6. Amygdala-hippocampal theta phase coherence for each condition. Theta phase coherence between pairs of electrodes targeting the amygdala (red dots) and hippocampal subfields (blue dots), depicted with hive plots for the aversive (a) and neutral (b) conditions. The magnitude of the phase locking value (PLV) between electrode pairs is presented in color, with warmer colors indicating a greater magnitude. Significance levels derived from permutation testing are indicated by the thickness of lines connecting each electrode pair.

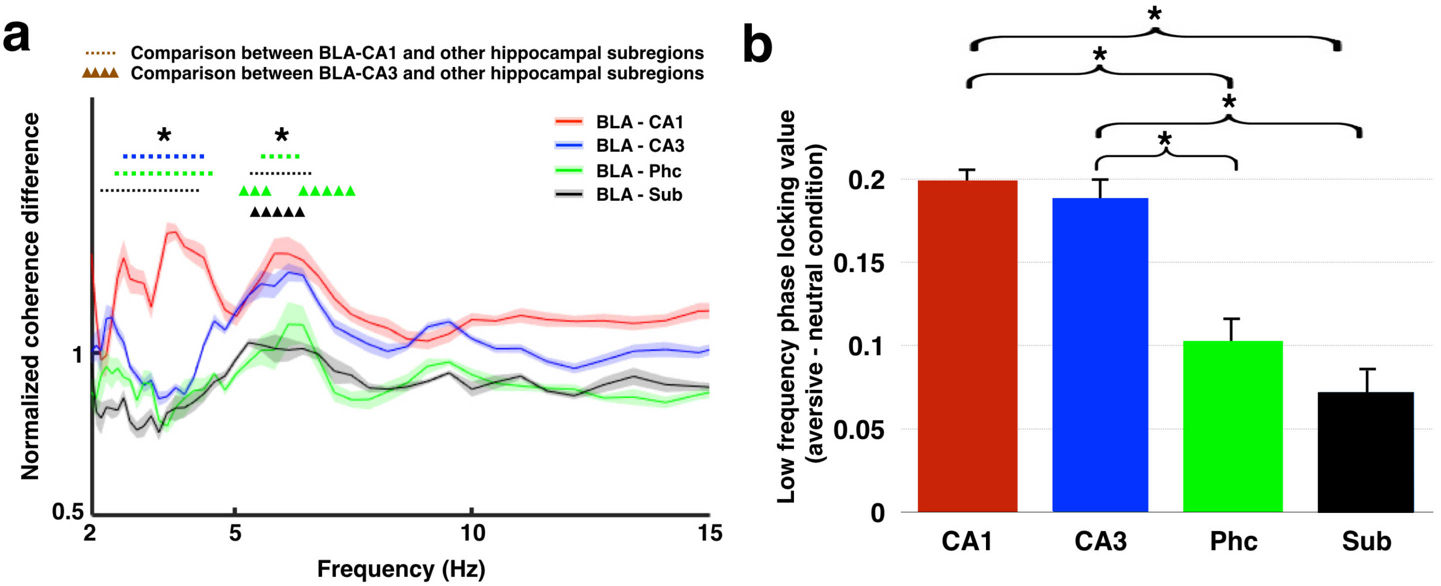

Supplementary Figure 7. Phase locking values (PLV) between basolateral amygdala (BLA) and hippocampal sub-regions. (a) Normalized coherence spectra averaged over all electrodes pairs between the BLA and the hippocampal subregions (CA1, CA3, Phc, and Sub). Increased theta PLV was observed in BLA-CA1 (red line) in contrast to BLA-CA3 (blue line), BLA-Phc (green line), and BLA-Sub (black line) electrode pairs when viewing aversive compared to neutral stimuli. The dots on top represent frequency ranges showing significant differences between BLA-CA1 and other electrode pairs ($p < 0.01$); triangles on the top denote significant differences between BLA-CA3 and other electrode pairs ($p < 0.01$). (b) PLV enhancement between BLA and hippocampal sub-regions when viewing the aversive vs. neutral stimuli among all 9 subjects. Error bars indicate ± s.e.m. across participants, and asterisk denotes significant differences at $p < 0.05$ with a post-hoc pairwise t-test (main effect for hippocampal sub-regions: $p = 0.004$). Phc = parahippocampal gyrus; Sub = subiculum.

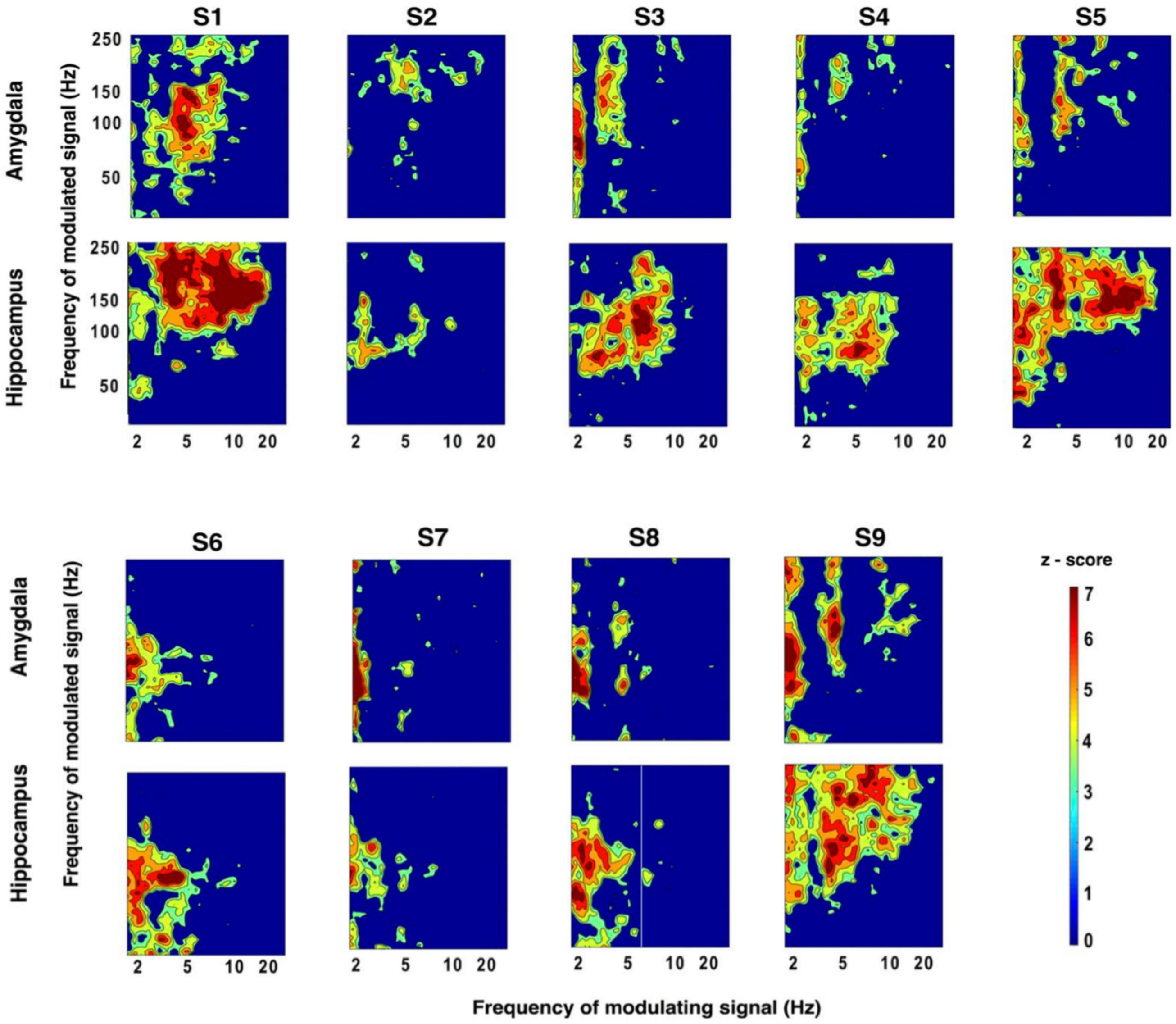

Supplementary Figure 8. Amygdala and hippocampus within electrode phase amplitude coupling (PAC). PAC comodulogram for differences between the aversive and the neutral condition is shown separately for the amygdala and hippocampus, with warmer colors denoting higher z-scores. Within each structure, increased PAC was observed when viewing the aversive compared to the neutral condition, with high gamma amplitude phase locked to phase of theta/alpha rhythms.

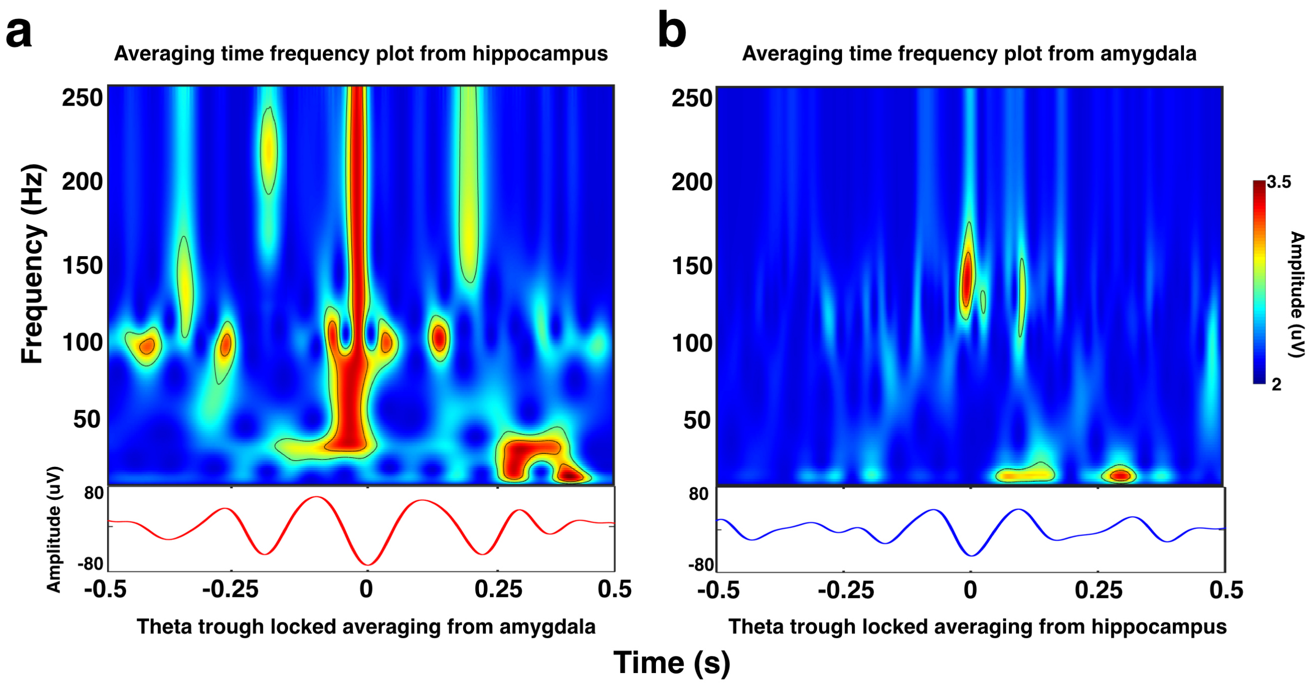

Supplementary Figure 9. Example of theta trough locked time frequency averaging for one subject in the aversive condition. (a) Normalized time-frequency average plot of mean power modulation from the hippocampus (upper panel) time-locked to the theta trough from the amygdala (lower panel). (b) Normalized time-frequency average plot of mean power modulation from the amygdala (upper panel) time-locked to the theta trough from the hippocampus (lower panel). Outermost contour in the time frequency plot indicates statistical significance ($p < 0.01$).

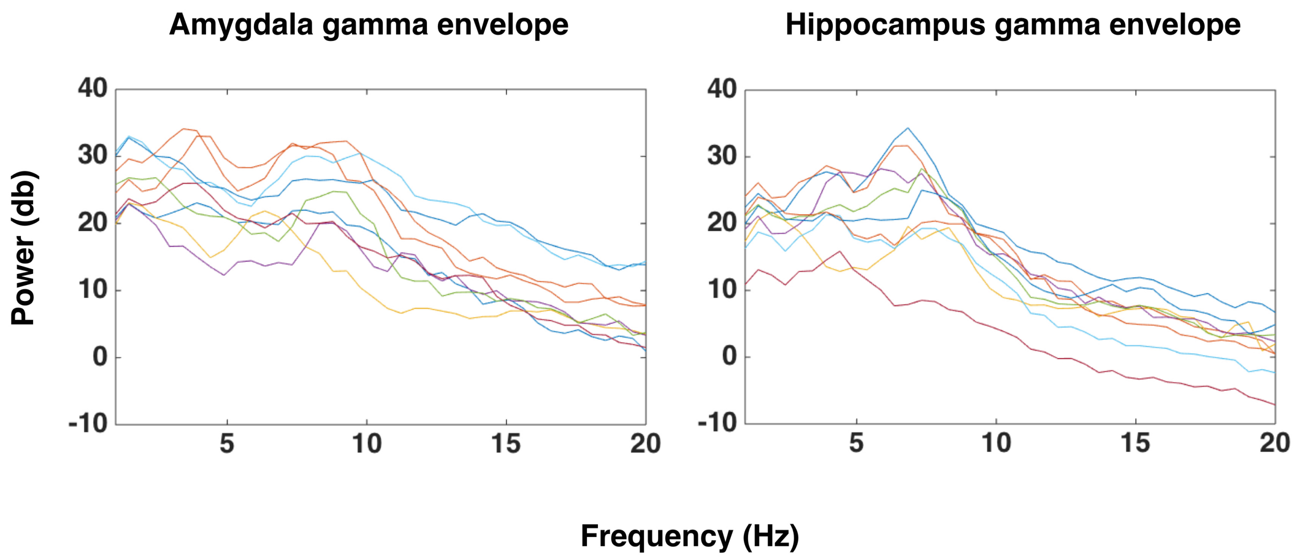

Supplementary Figure 10. Power spectral density (PSD) plots in a log-log (dB) scale for amygdala and hippocampus gamma envelope. PSD peaks around 7-8 Hz are evident across subjects in both regions. Each color line represents a single subject.

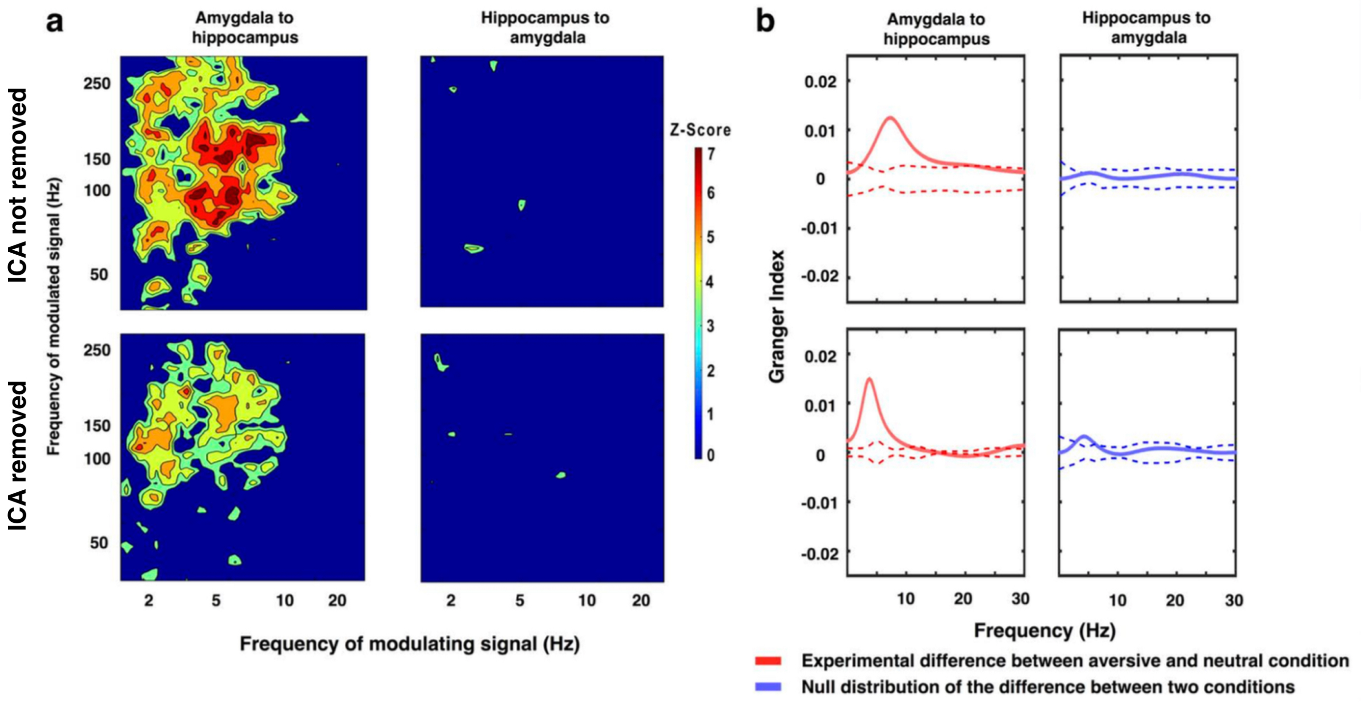

Supplementary Figure 11. Phase amplitude coupling and Granger causality with ICA correction for subject 9. Directional phase amplitude coupling (a) and Granger causality analysis (b) before and after independent component analysis (ICA)-based removal of eye movement artifacts. Significant amygdala-hippocampal directionality was evident before and after ICA-based artifact removal, demonstrating that eye movement artifact did not influence the results.

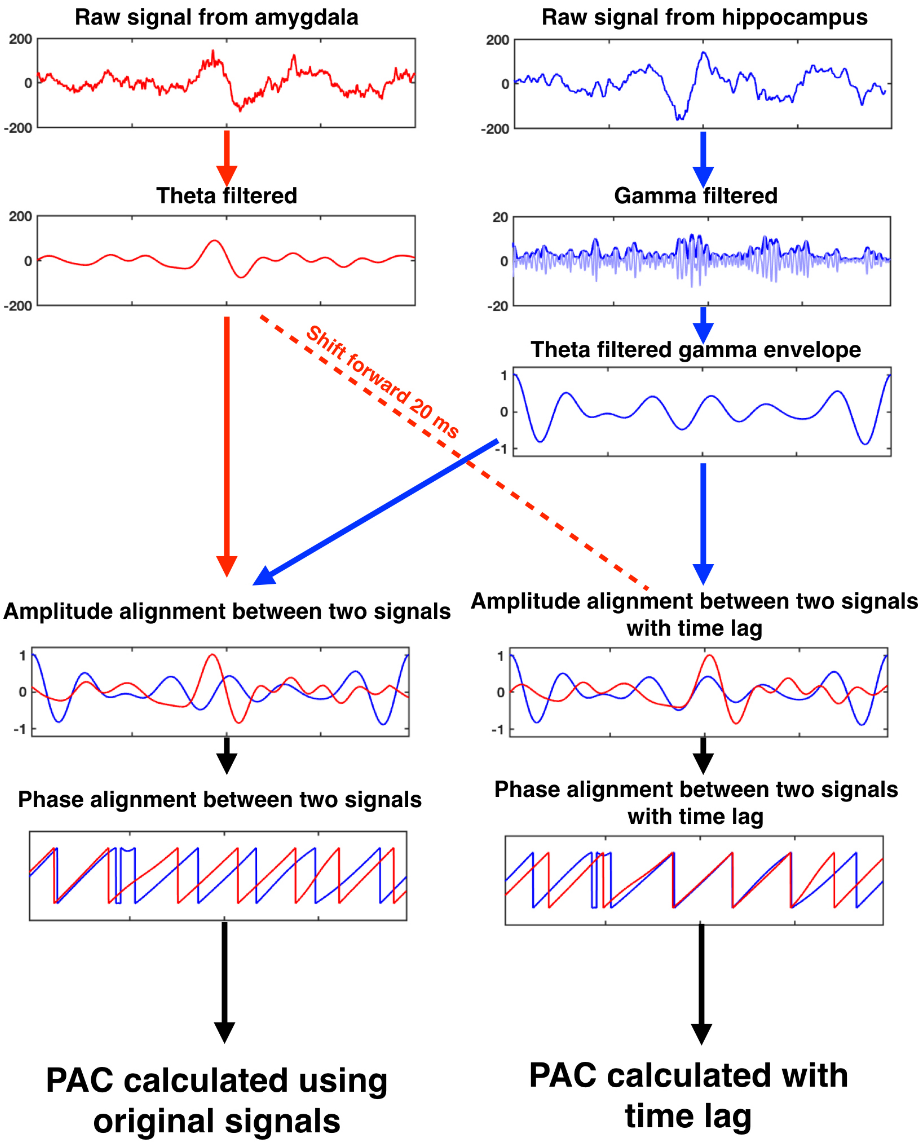

Supplementary Figure 12. Data processing schematic for calculating phase amplitude coupling (PAC) with time lag. The raw signal from the amygdala was band-pass filtered into a subject-specific theta/alpha component (5-9 Hz for the subject demonstrated here), while the raw signal from the hippocampus was band-pass filtered into high frequency gamma (70-180 Hz). We then extracted the amplitude of band-passed hippocampus high gamma and filtered this amplitude time series at the same theta/alpha band. The phase of both theta/alpha-filtered signal and the theta/alpha-filtered high gamma amplitude were extracted, and the PAC was calculated by computing the phase-locking between these two signals. By varying the time shift between two signals, the PAC can be then presented as a function of time (Figure 3b).

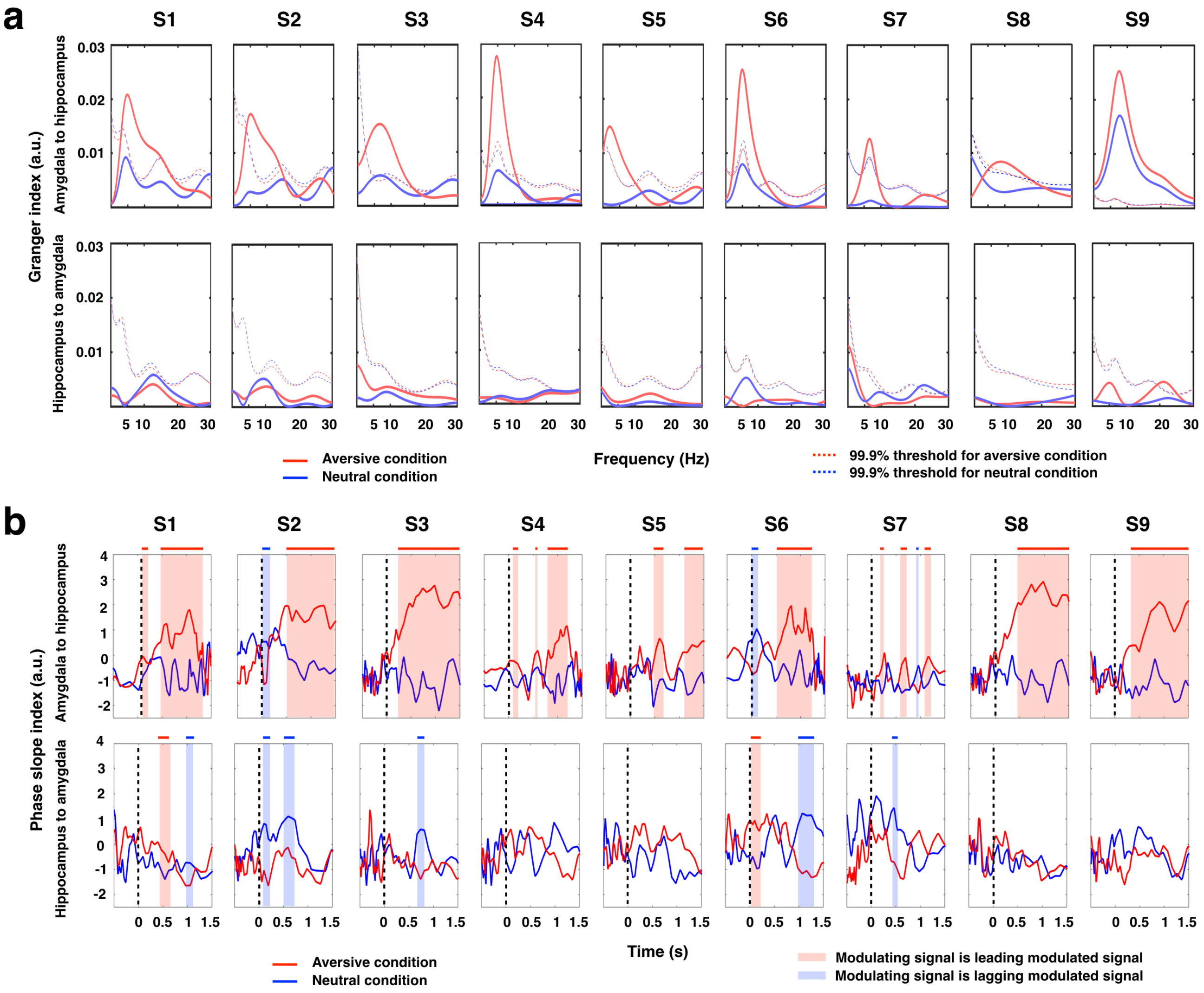

Supplementary Figure 13. Amygdala-hippocampus Granger causality and phase slope index for each subject. (a) Granger causality analyses demonstrated consistently stronger influence for the amygdala to hippocampus direction (top row) than for the hippocampus to amygdala direction (bottom row) when contrasting the aversive to the neutral condition for each subject. Solid lines are experimental data from the two conditions (red: aversive condition; blue: neutral condition). The 99.9% confidence intervals are presented as dashed lines. (b) Phase slope index (PSI) between the aversive and the neutral conditions calculated point-by-point across time using the subject specific theta band signal from the modulating channel (colored in red) and high gamma signal from the modulated channel (colored in blue) for each subject. Shaded regions denote significant differences between the two signals (all $p < 0.01$, permutation test), showing low frequency activity from the amygdala precedes hippocampus gamma for the majority of the stimuli duration.

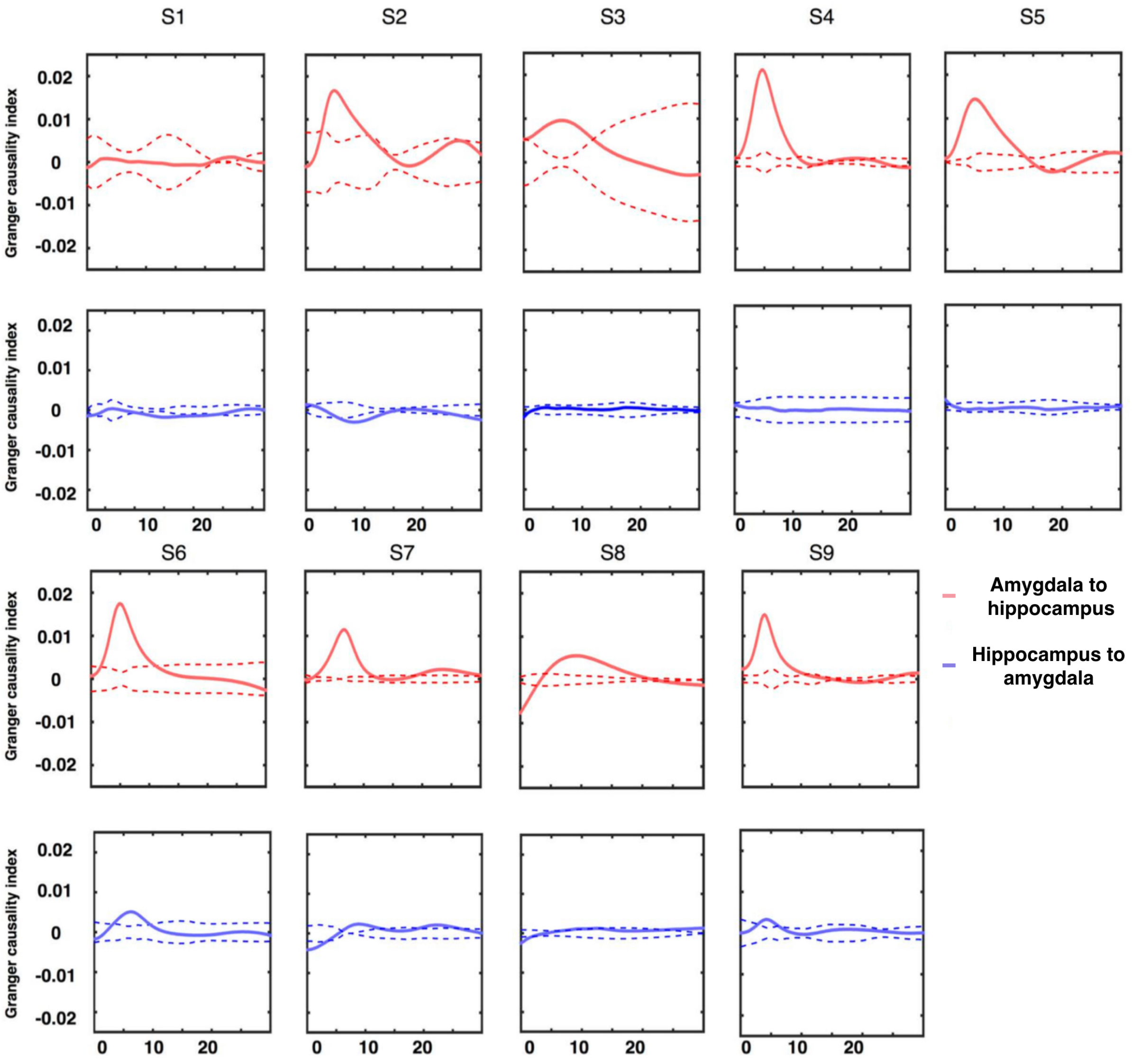

Supplementary Figure 14. Granger causality difference between aversive and neutral condition. Granger causality analyses demonstrated consistently stronger influence for the amygdala to hippocampus direction (red lines) than for the hippocampus to amygdala direction (blue lines) when contrasting the aversive to the neutral condition in all individuals except for subject 1. The solid lines represent the experimental data (the difference in Granger causality between the two conditions). The 99.9% confidence intervals for the null distribution are presented as dash lines.
